# An integrated analysis of rare CNV and exome variation in Autism Spectrum Disorder using the Infinium PsychArray

**DOI:** 10.1038/s41598-020-59922-3

**Published:** 2020-02-21

**Authors:** Elena Bacchelli, Cinzia Cameli, Marta Viggiano, Roberta Igliozzi, Alice Mancini, Raffaella Tancredi, Agatino Battaglia, Elena Maestrini

**Affiliations:** 1 Department of Pharmacy and Biotechnology, University of Bologna, Bologna, Italy; 2 IRCCS Stella Maris Foundation, Viale del Tirreno 331, 56128, Calambrone, Pisa, Italy

**Keywords:** Genetic predisposition to disease, Autism spectrum disorders

## Abstract

Autism spectrum disorder (ASD) is a neurodevelopmental condition with a complex and heterogeneous genetic etiology. While a proportion of ASD risk is attributable to common variants, rare copy-number variants (CNVs) and protein-disrupting single-nucleotide variants (SNVs) have been shown to significantly contribute to ASD etiology. We analyzed a homogeneous cohort of 127 ASD Italian families genotyped with the Illumina PsychArray, to perform an integrated analysis of CNVs and SNVs and to assess their contribution to ASD risk. We observed a higher burden of rare CNVs, especially deletions, in ASD individuals versus unaffected controls. Furthermore, we identified a significant enrichment of rare CNVs intersecting ASD candidate genes reported in the SFARI database. Family-based analysis of rare SNVs genotyped by the PsychArray also indicated an increased transmission of rare SNV variants from heterozygous parents to probands, supporting a multigenic model of ASD risk with significant contributions of both variant types. Moreover, our study reinforced the evidence for a significant role of *VPS13B, WWOX, CNTNAP2, RBFOX1, MACROD2, APBA2, PARK2, GPHN*, and *RNF113A* genes in ASD susceptibility. Finally, we showed that the PsychArray, besides providing useful genotyping data in psychiatric disorders, is a valuable and cost-efficient tool for genic CNV detection, down to 10 kb.

## Introduction

Autism spectrum disorders (ASDs) are a heterogeneous group of neuropsychiatric conditions characterized by impairments in social communication, as well as the presence of restricted interests, stereotyped and repetitive behaviors. ASDs have a worldwide prevalence of about 1%, with males about four times more likely to be affected than females^1^.

ASDs are highly heterogeneous, both in clinical presentation and with reference to the complex risk architecture. ASD individuals often display other psychiatric and medical conditions including intellectual disability (ID), epilepsy, sleep disorders, motor deficits (hypotonia, apraxia or motor delay), attention-deficit hyperactivity disorder (ADHD), and gastrointestinal disturbances.

Undoubtedly, genetic factors play a substantial role in ASD risk. With the availability of microarray and massively parallel sequencing platforms, significant progresses have been made in the last decade in elucidating the underlying genetic risk factors. There is a rising awareness that both common and rare variants contribute to ASD risk. Despite the accumulating evidence supporting a major role of common genetic variation in ASDs^2^, the relative risk conferred by each common variant is very low, thus the identification of common risk variants robustly associated to ASD requires very large sample sizes, which are just starting to be attainable thanks to massive international efforts. A recent genome-wide association meta-analysis of 18,381 ASD cases and 27,969 controls first reported common risk variants reaching genome-wide significance^3^.

Given the difficulties in the identification of common risk alleles, most of our current knowledge in ASD genetics comes from the analysis of rare variants, which typically confers a much higher risk in a single individual. Large-scale genomic studies have established the role of *de novo* and rare inherited copy-number variants (CNVs) and protein-disrupting single-nucleotide variants (SNVs) in ASD pathogenesis. In particular, rare penetrant genic CNVs are thought to increase the risk of having ASD in 5–10% of the individuals, depending on the cohort examined. Several of these rare CNVs are recurrent and associated with other neuropsychiatric conditions, some inherited from apparently unaffected parents and none individually account for more than 1% of ASD cases, leading to the identification of hundreds of candidate genes. Particularly convincing is the role of *de novo* CNVs, with recent estimates that ∼80% of the individuals with a *de novo* CNV and an ASD diagnosis would not be affected if they did not have the CNV^2^, but genomic studies also suggested a role for inherited CNVs with lower penetrance^4–6^.

Here, we present a genome-wide CNV analysis of 127 Italian ASD families genotyped with the PsychArray, a SNP array developed by Illumina in collaboration with the Psychiatric Genomic Consortium (PGC). The PsychArray contains a genome-wide backbone of approximately 270,000 tag SNPs, 250,000 rare and low-frequency exonic variants, and approximately 50,000 custom markers selected based on evidence from prior genetic studies of psychiatric disorders, including ASD. Here we show that, despite the rather low genome-wide SNP density, the PsychArray allows a reliable detection of genic CNVs down to 10 kb. In addition, the exome and rare variant content of this SNP array allows the identification of an interesting subset of rare genic variants. The main aim of our study was thus to conduct an integrated analysis of both CNV and SNV data in a clinically well-defined collection of ASD Italian families.

At present, whole genome “chromosomal microarray” (CMA) is recommended as a first tier clinical genetic test for detecting disease-causing CNVs in individuals with ASD or other neurodevelopmental disorders^7–9^. However, it can sometimes be difficult to establish or exclude the clinical relevance of specific CNVs, as most CNVs are characterized by incomplete penetrance and variable expressivity. Accumulation of genotype-phenotype information is therefore essential to consolidate known ASD loci and to identify novel candidate genes, with the aim of expanding our knowledge of ASD’s genetic background and improving the clinical care of people with ASD. A secondary aim of our study was thus to provide detailed clinical data of individuals carrying known causal CNVs and/or variants.

## Results

### Rare CNV burden analysis

**Figure 1:**
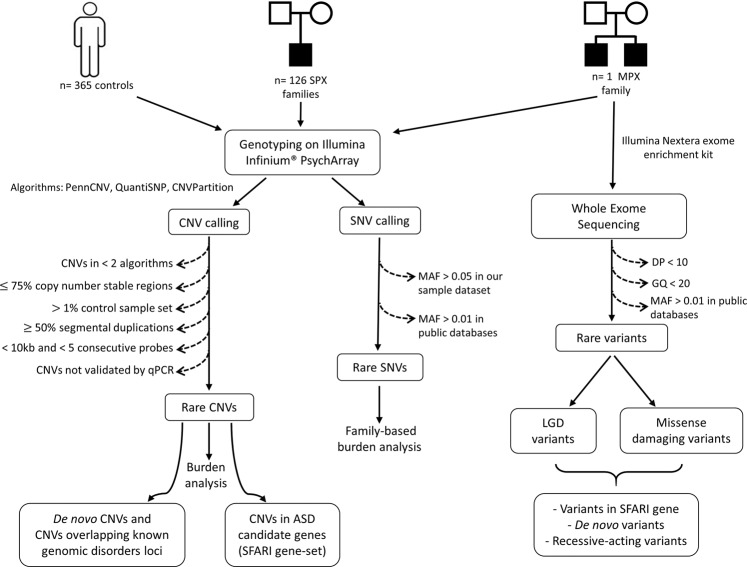
Schematic of the experimental design.

The characteristics of our clinical sample are reported in Table 1. All DNA samples, including 128 ASD individuals from 127 pedigrees, 238 parents and 365 controls were genotyped using the Illumina Infinium® PsychArray (Fig. 1).

**Table 1:** Summary of the clinical and diagnostic characteristics of the ASD sample.

	*n*	All sample (n = 128)			*n*	Males (n = 106)			*n*	Females (n = 22)		
		Mean	SD	Range		Mean	SD	Range		Mean	SD	Range
**ADI-R (diagnosis)**^**a**^												
Autism (3/3)^b^	48	−	−	−	35	−	−	−	13	−	−	−
Not autism (2/3)^c^	7	−	−	−	7	−	−	−	0	−	−	−
**ADI-R (score)**^**a**^												
Social interaction	55	17.5	5.0	8–28	42	17.5	5.3	8–28	13	17.5	4.1	12–25
Communication and Language	55	11.8	4.6	5–23	42	12.0	4.4	5–23	13	11.4	5.4	7–22
Restricted and repetitive behaviours	55	5.9	2.3	2–12	42	6.0	2.4	2–12	13	5.3	2.0	3–9
**ADOS (diagnosis)**^**d**^												
Autism^e^	56	−	−	−	43	−	−	−	13	−	−	−
Autism Spectrum^f^	30	−	−	−	26	−	−	−	4	−	−	−
Non spectrum^g^	6	−	−	−	4	−	−	−	2	−	−	−
**ADOS (score)**^**d**^												
Communication domain	83	4.5	1.9	0–11	65	4.4	1.9	0–11	18	4.7	2.2	1–8
Social domain	83	8.2	2.9	3–14	65	8.1	2.7	3–14	18	8.6	3.7	4–14
Social plus communication	88	12.7	4.2	5–22	70	12.6	3.9	5–22	18	13.3	5.6	6–22
Stereotypic behavior	68	2.4	1.8	0–7	55	2.2	1.6	0–7	13	3.2	2.4	0–6
Play	64	2.2	1.4	0–6	50	2.2	1.4	0–6	14	2.6	1.2	0–4
**Language level (ADOS module)** ^**h**^												
non verbal- single/some words (mod 1)	57	−	−	−	44	−	−	−	13	−	−	−
phrase or fluent speech (mod 2/3/4)	31	−	−	−	26	−	−	−	5	−	−	−
**VABS (age equivalents)** ^**i**^												
Age at VABS administration (months)	108	80.10	35.90	27–186	87	81.70	36.28	27–186	21	73.48	34.62	37–160
**Communication domain (months)**	104	45.05	34.48	18–147	83	47.37	35.21	18–147	21	35.86	30.50	18–142
Receptive (months)	105	33.05	30.08	18–194	84	35.08	32.27	18–194	21	24.90	17.15	18–81
Expressive (months)	104	41.08	30.70	18–120	83	43.00	31.33	18–120	21	33.48	27.43	18–120
Written (months)	105	55.81	28.93	37–157	84	57.55	29.60	37–157	21	48.86	25.58	37–140
**Daily living Skills domain (months)**	107	42.74	29.45	18–175	86	43.97	28.42	18–168	21	37.71	33.64	18–175
Personal (months)	107	38.80	25.22	18–133	86	39.65	25.29	18–133	21	35.33	25.21	18–124
Domestic (months)	104	56.98	33.26	34–203	83	58.05	32.41	34–203	21	52.76	36.96	34–203
Community (months)	105	42.81	26.96	26–168	84	44.42	27.34	26–168	21	36.38	24.94	27–141
**Socialization domain (months)**	106	31.65	17.87	18–130	85	33.58	19.20	18–130	21	23.86	6.98	18–37
Interpersonal relationship (months)	103	28.80	18.03	18–145	82	29.56	18.65	18–145	21	25.81	15.40	18–83
Play and Leisure time (months)	104	32.41	22.86	18–141	83	33.46	21.87	18–121	21	28.29	26.60	18–141
Coping skills (months)	103	45.00	18.33	18–125	83	45.77	17.88	18–122	20	41.80	20.24	32–125
**Motor skills domain (months)**	97	41.91	14.34	18–65	78	43.62	13.88	18–65	19	34.9	14.4	18–65
Gross Motor (months)	98	43.60	14.44	18–64	79	44.84	14.12	18–64	19	38.47	14.99	18–64
Fine Motor (months)	97	40.59	16.07	18–66	78	42.73	15.79	18–66	19	31.79	14.50	18–66
**Intelligence Quotient (IQ)**^**j**^												
Total IQ^k^	34	78.5	19.1	36–112	31	77.7	19.0	49–112	3	86.3	22.5	70–112
Verbal IQ^l^	35	81.0	20.0	43–124	31	79.6	20.0	47–124	4	91.8	19.3	75–109
Non Verbal IQ^m^	75	84.6	22.9	31–135	63	86.0	22.5	39–135	12	77.3	24.7	31–113
**Developmental level (GMDS)**^**n**^												
Global Developmental Quotient (DQ)	20	57.8	17.0	31–79	17	59.6	17.4	31–79	3	47.3	11.7	37–60
A - Locomotor	11	77.1	16.9	45–109	10	78.2	17.4	45–109	1	66.0	−	−
B - Personal-Social	12	52.6	15.0	28–77	9	58.1	12.5	31–77	3	36.0	7.5	28–43
C - Hearing and language	23	40.5	18.9	10–73	19	44.2	18.4	17–73	4	23.0	9.8	10–31
D - Eye and Hand Co-ordination	13	56.2	23.8	28–96	10	62.2	23.8	29–96	3	36.0	7.5	28–43
E - Performance	29	66.2	22.9	29–107	23	69.5	22.4	29–107	6	53.5	21.9	36–93
F - Practical Reasoning	2	67.5	17.7	55–80	2	67.5	17.7	55–80	0	−	−	−
**Child Behavior Checklist (t-scores)**												
Internalize problems	97	63.8	8.8	45–86	79	63.1	8.9	45–86	18	66.7	7.5	49–78
Externalize problems	97	57.8	9.7	33–77	79	57.6	9.9	33–77	18	58.6	8.6	44–74
Total problems	97	63.2	9.7	38–89	79	62.6	9.9	38–89	18	65.5	8.4	46–77

^a^Autism Diagnostic Interview – Revised; ^b^Probands met all 3 criteria for an ASD diagnosis based on ADI-R; ^c^Probands 2/3 criteria for ASD diagnosis based on ADI-R: they have a diagnosis of ASD based on the other assessments and/or DSM criteria; ^d^Autism Diagnostic Observation Schedule (ADOS-G or ADOS-2); ^e,f^Probands met criteria for a diagnosis of autism or autism spectrum based on ADOS; ^g^Probands did not meet criteria for an autism/ASD diagnosis based on ADOS: they have a diagnosis of ASD based on the other assessments and/or DSM criteria; ^h^Language level deduced from the chosen module for the ADOS observation; ^i^Vineland Adaptive Behavior Scale; ^j^Cognitive assessment is available for children in whom a standardized test could be administered. The test choice was based on age, language level and individual characteristics of the subject; ^k^Average of the total IQ scores obtained from the assessments of children who were given WPPSI-III, WISC (R, III or IV), KBIT-2 scales; ^l^Average of the verbal IQ scores obtained from the assessments of children who were given WPPSI-III, WISC (R, III or IV), KBIT-2 scales; ^m^Average of the non verbal IQ scores obtained from the assessments of children who were given WPPSI-III, WISC (R, III or IV), KBIT-2 or Leiter-R scales; ^n^Griffiths Mental Development Scales.

To our knowledge, only a few studies reported the use of PsychArray data for genome-wide CNV detection^10–12^. Hence, we established a CNV calling protocol based on three different CNV detection algorithms and set criteria to define stringent CNV calls (Supplementary Methods). To test the reliability of our protocol we tested 31 CNVs for validation using qPCR (Supplementary Table S1 and Supplementary Fig. S1). All tested CNV were validated, demonstrating the specificity of our algorithm of this cost-effective array in detecting small genic CNVs, down to approximately 10 Kb. To test the sensitivity of the PsychArray for genic CNV detection and our analysis protocol, we have included an ASD sample with a 15q13.3 duplication, spanning a region of approximately 500 kb including the entire *CHRNA7* gene, that was previously genotyped with the high-density array Illumina 1M-duo array^5^. As expected, the PsychArray wasn’t able to detect most intragenic CNVs and CNVs spanning segmental duplication regions called by the Illumina 1M-duo array, due to the lack of probes in those regions; conversely, it allowed the identification of all rare genic CNVs called by the 1M-duo array, including the clinically relevant *CHRNA7* duplication (see Supplementary Table S2).

According to our stringent criteria, a total of 253 rare CNVs (< 1% frequency) were detected among 128 ASD cases, while 639 rare CNVs were identified in 363 controls (Supplementary Table S3).

To test the impact of rare CNVs in cases and controls, we performed a global CNV burden analysis. As shown in Table 2, there is a higher proportion of cases who have at least one rare CNV event compared to controls (0.90 versus 0.82, empirical *p* = 0.019), and this difference is more significant if we consider only deletions (0.66 versus 0.54, empirical *p* = 0.008). Moreover, there is a trend for an increased rate of rare CNVs in ASD cases compared to controls, and this increased rate becomes significant when only deletions are considered (1.05 versus 0.81, empirical *p* = 0.009).

**Table 2:** Rare CNV Burden Analysis in Cases and Controls.

	ASD cases (n = 128)	Controls (n = 363)	P value		ASD cases (n = 128)	Controls (n = 363)	P value
***All CNVs***				***Deletions***			
*N*	253	639		*N*	134	295	
RATE	1.977	1.76	0.073793	RATE	1.047	0.8127	**0.009399**
PROP	0.8984	0.8154	**0.019098**	PROP	0.6641	0.5372	**0.008099**
TOTKB	296.1	352.6	0.823018	TOTKB	166.4	226.2	0.815718
AVGKB	143.5	156.3	0.679432	AVGKB	112.9	144	0.720728
***SFARI genes, all CNVs***				***SFARI genes, deletions***			
*N*	33	57		*N*	23	36	
GRATE	0.2578	0.157	**0.039896**	GRATE	0.1797	0.09917	**0.042196**
GPROP	0.1953	0.1295	0.050795	GPROP	0.1562	0.08264	**0.015199**

N: Number of events; RATE: Number of CNVs per person; PROP: Proportion of cases/controls to have at least one CNV; TOTKB: Total kb length spanned per person; AVGKB: Average segment size per person; GRATE: Number of SFARI genes spanned by CNVs per person; GPROP: Proportion of cases/controls to have CNVs spanning at least one SFARI gene.

To investigate if CNVs affecting genes previously associated with ASD mainly contribute to the observed burden results, we used the SFARI gene database (https://gene.sfari.org/) and its ranking system, restricting the analysis to the set of SFARI ASD genes (Supplementary Table S4). The number of SFARI genes affected by deletions and the proportion of subjects having at least one event are significantly higher in cases versus controls (Table 2). Notably, re-analysis of the deletion burden after removal of those deletions affecting SFARI genes lacked significance in both comparisons (GRATE *p* = 0.18, GPROP *p* = 0.16).

Finally to confirm the presence of a statistically significant enrichment for SFARI ASD candidate genes, we performed gene-set enrichment analysis controlling for case-control differences in CNV rate and size, showing a significant enrichment for the count of ASD candidate gene affected by a CNV (empirical *p* = 0.034) in cases versus controls.

### CNVs overlapping with known genomic disorders loci

In order to identify in our cohort CNVs of potential clinical significance, we sought CNV overlapping loci previously implicated in known genomic disorders (https://decipher.sanger.ac.uk/) and recurrent CNV shown to increase the risk of developing early-onset neurodevelopmental disorders^13^.

We identified 5 CNVs (2 deletions and 3 duplications) in regions known to associate with autosomal genomic disorders, most of which correspond to genomic hotspots flanked by segmental duplication (Table 3a). The two identified deletions are both *de novo*, while the 3 duplications are inherited from unaffected parents.

**Table 3:** CNVs overlapping with known genomic disorders loci (a) and *de novo* CNVs (b).

Cytoband	Coordinates min/max	length (bp) min/max	CN	Sample Sex	Inheritance	Genes	Freq^a^	Penetrance %^11^	Clinical Diagnosis^b^	Other rare genic CNVs
***a) CNVs Overlapping with Known Genomic Disorders***										
1p36.32	chr1:2473258-3118326	645069	1	AB27 Male	*De novo*	9 genes	0	1p36 del (100% penetrance in DD/ASD/CM)	PDD	
15q11.2	chr15:22755185-23228712/ chr15:22383300-23668092	473528/1284793	3	AB111 Male	Maternal	10 genes	4 (1TP, 2NP, 1 C)	15q11.2 dup (1.8% penetrance for ASD)^17^	PDD	
16p13.11	chr16:15493046-16301530/ chr16:15395596-16859425	808485/1463830	3	AB35 Female	Paternal	18 genes	2 (1TP, 1 C)	16p13.11 dup (8.4% penetrance in DD/ASD/CM)	PDD	(chr10:68065751-68180999)x1mat
17q12	chr17:34815551-36249430/ chr17:34461869-36455348	1433880/1993480	3	AB164 Male	Maternal	11 genes	1 (TP)	17q12 dup (17% penetrance in DD/ASD/CM)	PDD	(chr11:4387760-4409718)x3mat
22q13.33	chr22:50814075-51181759/ chr22:50764057-51304566	367684/540509	1	AB133 Female	*De novo*	19 genes	0	Phelan-McDermid del (100% penetrance in DD/ASD/CM)	ASD	(chrX:38490844-38624791)x3mat
***b) Rare De Novo genic CNVs***										
5p13.2	chr5:35730729-35991299	260571	3	AB161 Male	*De novo*	*CAPSL, IL7R, SPEF2, UGT3A1*	0		PDD	(chr16:3010466-3032566)x1mat; (chr1:236850052-237167218)x3pat
13q34	chr13:114323997-114475037	151041	1	AB84 Male	*De novo*	*FAM70B, GRK1, LOC100130386*	0		PDD	

^a^Frequency a in 363 controls (C) and 238 parents (P): TP: transmitting parent; NP: non-transmitting parent.

^b^PDD: pervasive developmental disorder according to the DSM-IV; ASD: autism spectrum disorder according to the DSM-5.

The female proband AB133 has a terminal *de novo* deletion involving the distal part of chromosome 22q13.33 that includes the *SHANK3* gene, considered the culprit gene for the Phelan-McDermid syndrome (PMS). PMS associated deletions vary in size from 45 kilobases (kb) to more than nine megabases (Mb) with a possible correlation between deletion size and presence and/or severity of some PMS symptoms^14^. The *de novo* 22q13.33 deletion identified in our ASD proband, presenting with absence of speech and ID, spans only about 530 kb.

A *de novo* 645 kb interstitial deletion encompassing the 1p36.32 locus was identified in the male proband AB27. Microdeletion 1p36 is a well characterized microdeletion syndrome associated with ID of variable degree and other clinical features, such as epilepsy and characteristic craniofacial features^15^. Interstitial deletions are present in about 29% of the patients, while approximately 52% have a *de novo* terminal 1p36 deletion^16^. The deletion reported here spans 9 genes from *TNFRSF14-AS1* to the first 2 coding exons of the *PRDM16* gene, and is among the smallest reported in the 1p36 locus. Our proband, with a high functioning ASD, had lax ligaments, deep-set eyes, and bilateral overfolded helix; but none of the distinctive clinical features of the “deletion 1p36” syndrome. Such findings confirm that the critical region for the core phenotype of the syndrome is toward the distal end of the short arm of chromosome 1 (1p36.33–1p36.32).

The male proband AB164, with ASD /ADHD, and borderline cognitive impairment, without dysmorphic features, carries a duplication on chromosome 17q12, inherited from an apparently healthy mother, overlapping the 17q12 recurrent duplication. This is consistent with the observation that, in approximately 90% of cases, the 17q12 duplication is inherited from a parent who is often minimally affected or phenotypically normal^17^.

A paternal inherited duplication encompassing the region 16p13.11 is present in the female proband AB35, with ASD, and mild cognitive impairment, without dysmorphic features, which overlaps with the likely core pathogenic region at the 16p13.11 locus (interval II) and includes the *NDE1* gene, which is the major candidate gene for the neurodevelopmental phenotypes associated with the 16p13.11 CNVs^18^.

Finally, a 15q11.2 duplication, overlapping the BP1-BP2 genomic interval was identified in male proband AB111, with ASD, mild cognitive impairment, without dysmorphic features. The BP1-BP2 CNVs has been shown to have a modest impact on ASD risk, however maternal duplications transmitted to male probands, as in our case, have been suggested to confer a greater effect on ASD-related phenotype^19^.

### *De novo* CNVs

Using the trio-based CNV calling algorithm implemented in PennCNV, and subsequent qPCR validation we identified 4 rare genic *de novo* CNVs: the two *de novo* deletions described above (Table 3a), and two additional *de novo* CNVs that are smaller, do not overlap known loci, and therefore have a more uncertain pathogenic role (Table 3b).

Case AB161, with ASD and borderline cognitive impairment, without dysmorphic features, has a *de novo* duplication on chromosome 5p13.2 including 4 genes, namely *CAPSL, IL7R, SPEF2, UGT3A1*. *SPEF2* is entirely duplicated; it encodes for Sperm flagellar protein 2, involved in cilia function^20^ and its transcript is a FMRP target^21^. Proband AB161 displayed two other inherited rare genic CNVs: a maternal deletion of 3 genes on chr16p13.3, and a paternal duplication on chromosome 1q43 including two genes: *ACTN2* and *MTR*. Interestingly, the *MTR* gene is another FMRP target gene and it has been implicated in methylcobalamin deficiency type G (MIM:250940), an autosomal recessive inherited disease that causes mental retardation, macrocytic anemia, and homocystinuria.

Male proband AB84, with ASD and a relative macrocephaly, carries a 151 kb de novo deletion on chromosome 13q34 including 3 genes. None of these genes has been previously implicated in neuropsychiatric disorders.

### Rare inherited copy number variants in ASD candidate genes

In addition to the CNVs overlapping known genomic disorders, we observed 24 inherited CNVs from 23 probands, intersecting ASD candidate genes. The clinical features of ASD probands and the details of the CNVs overlapping SFARI genes are reported in the Supplementary Table S5.

**Figure 2:**
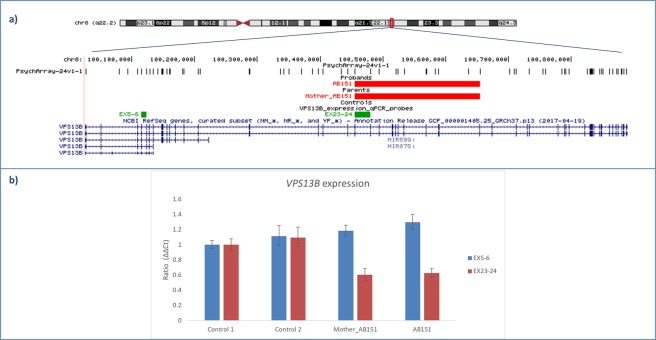
*VPS13B* deletion. (**a**) UCSC hg19 screenshot showing the 200 kb maternally inherited deletion impacting the *VPS13B* gene identified in case AB151. No CNVs in *VPS13B* have been detected in our control cohort. qPCR probes used to test *VPS13B* expression are shown in green; (**b**) *VPS13B* expression levels in the deletion carriers (AB151 and the mother of AB151) and in two controls.

Among these CNVs, a notable case finding is the identification of an intragenic multiexonic deletion of the *VPS13B* gene, classified as a syndromic ASD candidate gene in the SFARI database, in proband AB151 (Fig. 2). By real time qPCR we confirmed that the deletion is inherited from the unaffected mother and includes exons 23–35 of the *VPS13B* full-length isoforms (1–62 exons, NM_152564.4 and NM_017890.4). To assess if the identified deletion affects *VPS13B* expression, we performed a qPCR on cDNA samples of proband AB151 and his mother, and two controls. A significant reduced expression of *VPS13B* full-length isoforms was observed in the mother and in the proband, confirming that the deletion causes a decrease of *VPS13B* full-lenghth isoforms. The proband has ASD with an impaired adaptive functioning, and underwent surgery for trigonocephaly at age 4 months.

**Figure 3:**
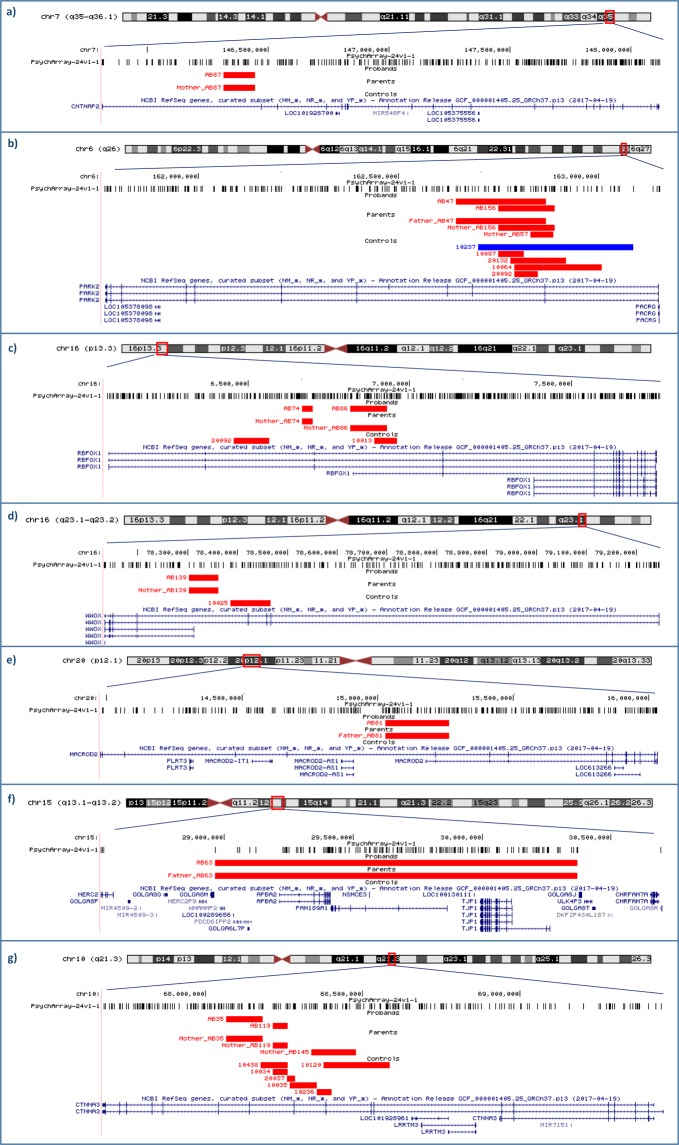
Most notable CNVs intersecting SFARI genes. UCSC hg19 screenshots reporting the most notable CNVs impacting SFARI genes identified in our ASD sample and in controls. PsychArray probes are shown. (**a**) A 128 kb maternally inherited intronic deletion in the *CNTNAP2* gene in case AB87. No CNVs in *CNTNAP2* have been detected in our control cohort; (**b**) *PARK2* CNVs in 2 cases (AB47 and AB156) and 5 controls; (**c**) *RBFOX1* deletions in 2 cases (AB74 and AB86) and 2 controls; (**d**) *WWOX* non overlapping deletions in case AB139 and in one control subject; (**e**) A 236 kb paternally inherited deletion in the *MACROD2* gene in case AB81. No CNVs in *MACROD2* have been detected in our control cohort; (**f**) A 1.4–2.2 Mb paternally inherited deletion mapping in 15q13.1-q13.2 locus and impacting at least nine genes, including *NDNL2* and *APBA2*. No CNVs in this locus have been found in our control sample; (**g**) *CTNNA3* deletions in 2 cases (AB35 and AB119), one non-transmitting mother (mother of case AB145) and 6 controls.

A second notable CNV is a *CNTNAP2* deletion in proband AB87, a well-established ASD candidate gene (SFARI score 2 S)^22^ (Fig. 3).

Furthermore, we identified 8 CNVs overlapping ASD genes with a suggestive evidence supporting their link to ASD (SFARI score = 3) and 14 CNVs intersecting genes implicated in ASD with minimal evidence (SFARI score = 4). The most notable CNVs in these groups included (Fig. 3):

- Two inherited deletions overlapping exon 2 and/or exon3 of the *PARK2* gene have been identified in cases AB47 and AB156.
- Two different maternally inherited deletions in *RBFOX1* have been identified in two ASD subjects: one in case AB74 including *RBFOX1* intron 2, the other one in proband AB86 encompassing the noncoding exon 1 of *RBFOX1* transcript variant 6 (NM_001142334.1).
- One maternally inherited deletion involving the last exon of the two shorter gene transcript variants of the *WWOX* gene (NM_130791.3 and NR_120436.1) has been identified in proband AB139.
- A paternally inherited deletion involving *MACROD2* exon 6 (NM_080676.5) has been identified in case AB81.
- A large paternally inherited deletion, located on the proximal region of the long arm of chromosome 15, was identified in case AB63. The minimal deleted region encompasses nine genes, including the two ASD candidate genes, *NDNL2* and *APBA2* (SFARI score = 4).
- Two maternally inherited deletions encompassing intron 11 and exon 12 of the *CTNNA3* gene have been identified in cases AB119 and AB35, respectively. It is worthy of note that case AB35 also carries a large paternally inherited duplication encompassing the region 16p13.11, associated with a wide range of neurodevelopmental disorders (Table 3a). Since we have previously implicated *CTNNA3* as a candidate gene in ASD acting in a recessive mode of inheritance^23^, we also investigated if the exonic deletion in case AB35 could act by unmasking rare variants in the non-deleted allele. However, sequence analysis of the entire *CTNNA3* coding sequence in case AB35 and in his parents, did not identify rare (MAF < 1%) exonic variants. We identified seven *CTNNA3* deletions in 6 controls and in one non-transmitting mother, consistent with the previously reported evidence that heterozygous deletions in *CTNNA3* are not pathological^23^.

### Rare genic SNV analysis

Leveraging the presence of probes for over 250,000 for putative functional exonic variants on the Psych-array, as well as 50,000 markers selected based on evidence from prior genetic studies of psychiatric disorders, we used genotyping data to perform an association analysis focused on rare genic variants.

Since single variant analysis was unfeasible due to lack of power, we carried out a global burden analysis. In order to avoid the problem of cryptic stratification between cases and controls, that is particularly relevant in rare variants analysis because the spectrum of rare variation can differ greatly between populations, we adopted a more robust family-based approach. After pruning for variants in linkage disequilibrium (LD), we tested the total burden of 51,547 genic rare variants using an extension of the transmission disequilibrium test (TDT-BRV)^24^, that revealed a significant excess transmission for the rare alleles from heterozygous parents to affected offspring (129,177 transmitted versus 127,906 untransmitted rare alleles, TDT p = 0.0122).

### Exome sequencing

In the single multiplex family included in our study (AB162/AB163), CNV analysis did not detect any potentially relevant CNV. Thus, exome sequencing was performed to test if point mutations might explain the ASD phenotype shared by the two affected brothers. We prioritized 39 exonic rare variants predicted deleterious and belonging to one of three “high-risk” categories: (i) SFARI gene-set; (ii) recessive-acting variants; (iii) *de novo* variants (Supplementary Table S6). We observed that about 56% (22 out of 39) of the prioritized rare variants have been also genotyped in the PsychArray. This provided useful information about the frequency of these variants in the entire sample of cases and controls drawn from the same population.

We identified 30 Likely Gene Disrupting (LGD) or damaging missense variants in ASD candidate genes, among which 10 are shared by both affected siblings. We highlight a shared damaging missense mutation, affecting *GPHN*, a neuronal synaptic gene predicted to be haploinsufficient (pLi score = 0.999981)^25^.

Moreover, we identified four X chromosome hemizygous damaging missense variants affecting the *FAAH2, RNF113A, SHROOM2, MID1IP1* genes, shared by the two affected brothers. Both nonsynonymous variants in *RNF113A* and *MID1IP1* were identified in another ASD family and were never found in our control cohort. The *FAAH2* missense variant was more frequent in cases versus controls (Fisher *P* = 0.00085). Thus, these X-linked variants could contribute to ASD susceptibility.

Finally, we discovered a *de novo* frameshift variant in the *TRPV1* gene, present in only one of the affects sibs (AB162). This 1bp-deletion in the *TRPV1* gene causes the introduction of 2 novel amino acids followed by a premature termination codon, probably inducing nonsense-mediated-decay.

## Discussion

Considerable advances have been made, over the past two decades, in understanding the genetic architecture of ASD. While a substantial proportion of ASD heritability is explained by common polygenic variation, rare variants have been shown to account for a considerable proportion of ASD cases, thus offering distinctive opportunities to understand ASD-related biology. In particular, the number of genes found to confer ASD liability has dramatically increased thanks to the identification of highly penetrant rare variants through high-resolution microarrays and exome/genome sequencing studies.

The main aim of this study was to analyze rare genetic variants in a homogeneous cohort of ASD families of Italian origin, in order to assess their contribution to ASD risk. Specifically, we focused on CNVs as they exert a more direct gene dosage impact and have been generally implicated in psychiatric diseases with larger effect size compared to SNPs. Moreover, we tested a set of exonic rare single nucleotide variants included in the Illumina PsychArray, enriched for variants previously implicated in neuropsychiatric disorders.

It is well-known that CNV detection using SNP-arrays varies widely in number of CNV calls, CNV size range, percentage of non-validated CNVs, and CNV type (exonic vs non-genic CNVs) across different array platforms. Therefore, the advantages and the limitations of each array should carefully be weighted, for an appropriate array selection. We chose the Illumina PsychArray for several reasons. First, even if this array has a lower genome-wide coverage in comparison to other high-density arrays, we demonstrate that the specificity of the detection of genic CNV using this array is very high. According to our experimental validation results, PsychArray data analyzed with our CNV calling algorithm and fulfilling criteria for stringent CNV calls, led to specific detection of genic CNVs down to 10 kb. Second, the Illumina PsychArray is enriched for exome variants and particularly protein altering variants implicated in psychiatric disorders and segregating at low allele frequencies in the genome. Therefore, this array offers an economical method to test the aggregate effect of rare variants in specific genes or gene sets. Obviously, a disadvantage of the PsychArray is the limited coverage of intergenic regions; however, we prioritized genic CNVs as they are generally more directly linked to pathogenic effects, while the interpretation of CNVs in non-genic regions still remains a challenging task. Another potential limitation of the PsychArray is its lower sensitivity compared to arrays with higher SNP coverage, as reported in a previous study that compared the performance of different arrays for genome-wide CNV detection^11^. However, the low sensitivity is likely to be confined to the detection of non-genic CNVs, given the reduced SNP coverage in intergenic regions. Moreover, in our study, a combination of multiple CNV detection algorithms was applied in order to obtain sensitive and reliable CNV calls, while the above-mentioned study applied only one analysis software at a time^11^. In order to test the PsychArray sensitivity in genic regions, we compared CNV calls detected by the PsychArray with calls from a high-resolution array in an ASD sample genotyped by both arrays. The PsychArray allowed the identification of all rare genic CNVs, including a clinically relevant *CHRNA7* duplication. Therefore, the false negative rate of genic CNV detection is not likely to represent a relevant issue for the aim of this study; in particular, given the PsychArray enrichment for probes in genes previously involved in psychiatric disorders, CNVs spanning ASD relevant genes are expected to be identified with a higher sensitivity.

However, the uneven distribution of SNPs and the requirement of an accurate multi-algorithms analysis currently prevent the application of the PsychArray in clinical practice, where the use of more robust and standardized microarrays is needed.

Our genome-wide CNV analysis identified 5 large clinically relevant CNVs, overlapping loci previously implicated in known genomic disorders. Among them, the two identified deletions are *de novo* and therefore more likely to be pathogenic^7^. Instead, the three recurrent large duplications exhibit reduced penetrance, as they are inherited from a phenotypically normal parent and, in two cases, they were also identified in our control sample (Table 3a). Consistent with an oligogenic CNV model, it is likely that these primary recurrent CNVs interact with other etiologic risk variants at other loci to exert phenotypic effect. Variants in the genetic background can indeed modulate the effects of recurrent CNVs, ultimately defining the phenotypic trajectory in CNV carriers, as it has been recently demonstrated for 16p11.2 deletion carriers^26^.

We also exploited CNV data to assess the aggregate effect of CNVs in cases and controls. Consistent with previous studies^5,27,28^, we found an increased global rare CNV burden in cases compared to controls, more notably for deletions. Furthermore, we identified a significant enrichment of rare CNVs intersecting ASD candidate genes. Notably, further support to the role of rare variants in ASD susceptibility came also from the family-based analysis of genic rare SNVs genotyped by the Illumina PsychArray.

Next, we took into consideration individual CNVs identified in our cohort, in order to investigate their potential relevance to the ASD phenotype based on gene constrain measures, CNV frequency in cases and controls, CNV location in the gene and relevant literature. 9 CNVs overlapping genes *VPS13B, WWOX, CNTNAP2*, *RBFOX1, MACROD2, APBA2*, and *PARK2* were of particular interest in our cohort (Supplementary Table S5).

Mutations in *VPS13B* have been associated with Cohen syndrome, a rare autosomal recessive neurodevelopmental disorder, and recessive variants have been reported in cases of ASD or ID with autistic features^29,30^. We showed that the inherited deletion identified in case AB151 causes a significant reduced expression of *VPS13B* full-length isoforms implicated in Cohen syndrome^31^, supporting the hypothesis that genes known to cause severe syndromes when completely knocked-out, might determine milder ASD phenotypes when only partially inactivated^29^.

Consistent with the above model, we identified an intragenic deletion of *WWOX* in case AB139, with a high-functioning ASD. Biallelic mutations in *WWOX* are responsible for early infantile epileptic encephalopathy-28 (EIEE28; OMIM 616211) and autosomal recessive spinocerebellar ataxia-12 (SCAR12; OMIM 614322). Rare CNVs overlapping *WWOX* have been reported at greater frequency in ASD cases versus unaffected controls^6^, thus suggesting that *WWOX* heterozygous variants act as weak risk factors, generally associated with milder ASD phenotypes, as in our case.

A third syndromic gene affected by a deletion in our sample is *CNTNAP2*. Homozygous or compound heterozygous mutations in *CNTNAP2* are the cause of cortical dysplasia-focal epilepsy syndrome (CDFES; OMIM 610042) and this gene has been implicated in multiple neurodevelopmental disorders, including autism and ID^22^. The inherited deletion identified in proband AB87 maps in *CNTNAP2* intron 1, without affecting the previously described binding site for the transcription factor *FOXP2* that should regulate its expression^32^. A recent comprehensive cross-disorder analysis of *CNTNAP2* role in psychiatric disorders^33^ suggested that *CNTNAP2* is unlikely to be a primary risk gene for psychiatric disorders, however this CNV might contribute to ASD risk interacting with other rare or common variants.

*RBFOX1* regulates alternative splicing events of genes critical for neuronal development and it has been strongly implicated in the etiopathogenesis of a wide spectrum of neurodevelopmental disorders including ASD. In particular, *RBFOX1* CNVs have been highlighted in individuals with ASD and other neuropsychiatric disorders;^34,35^ transcriptomic analyses of autistic postmortem brains revealed a reduced expression of *RBFOX1* in ASD patients which possibly results in altered splicing of *RBFOX1* target exons in synaptic genes^36^. In our study we identified two maternally inherited *RBFOX1* deletions in cases and two in controls. A similar *RBFOX1* deletion was reported in an autism family by whole genome sequencing in combination with a rare *NTM* deletion inherited from the other parent^37^. Interestingly, our proband AB86 also carries a paternal duplication in the ASD candidate gene *SNTG2*, supporting an oligogenic model for ASD risk in this family.

*MACROD2* gene has been implicated in ASD susceptibility by both CNVs and GWAS studies^3,38,39^. Moreover, a SNP in *MACROD2* was significantly associated with autistic-like traits in general population^40^. Therefore, the exonic *MACROD2* deletion in case AB81 is of particular interest, especially given the absence of *MACROD2* CNVs in our control sample, as well as in the CNV map of the human genome^41^.

CNVs at 15q13.1-q13.2 locus are likely due to non-allelic homologous recombination (NAHR) events between segmental duplications in proximal chromosome 15q breakpoints 3 and 4 (BP3–BP4 CNVs). Unlike the more common clinically relevant BP1–BP3 CNVs (PWS/AS critical region) and BP4–BP5 CNVs (15q13.3 microdeletion syndrome), BP3–BP4 CNVs have unclear significance given their rarity and the imperfect segregation with disease status in families with ID and/or developmental delay^42,43^. However, the presence of common clinical features among cases with BP3–BP4 deletions and the presence of genes with roles in development and nervous system function in the deletion region, suggest that this deletion may have a role in abnormal phenotypes in some individuals^44^. The most notable gene included in case AB63 deletion is *APBA2*, encoding a neuronal adapter protein essential for synaptic transmission^45^. Of note, our patient had hypotonia particularly involving the facial muscles.

CNVs in *PARK2* have been described in individuals with ASD and other neurodevelopmental disorders^46–48^ suggesting their pathogenic role in multiple brain processes. However, *PARK2* CNVs have also been found in control populations and an accurate analysis of the CNV location showed that, while CNVs targeting *PARK2* exons 5–12 are significantly more frequent in neurodevelopmental disorders cases than in controls, CNVs involving exons 2–4 are well-tolerated and very common both in cases and controls^48,49^. All *PARK2* deletions identified in our study map between exons 2 and 3 and their frequency is the same in cases and controls, suggesting that they do not represent major risk factors for ASD in these two ASD families.

The multiplex family included in our study (AB162/AB163) was further investigated by exome sequencing. The most interesting WES finding is an inherited missense variant in the *GPHN* gene, which is shared by both affected siblings. *GPHN* is a haploinsufficient gene encoding a key scaffolding protein in the neuronal postsynaptic membrane, with well-established functional links with synaptic proteins implicated in ASD, such as neuroligins and neurexins^50^. Moreover, *GPHN* deletions have been identified in ASD subjects and have been reported to be associated to a diverse range of neurodevelopmental conditions^50,51^. Among the chromosome X hemizygous variants shared by both affected brothers, we highlighted a missense variant in the FMRP-target gene *RNF113A*^21^. Interestingly, a *RNF113A* stop-gain mutation has been associated with an X-linked form of trichothiodystrophy (TTD), a disease characterized by a wide range of clinical features, including intellectual and developmental disabilities^52^. Finally, we identified a *de novo* frameshift variant in the *TRPV1* gene, only present in case AB162. *TRPV1* is a non-selective cation channel highly expressed in the brain, where it participates in several synaptic functions, such as modulation of spine morphology, synaptic transmission and plasticity^53^. *TRPV1* plays an important role in the transmission and modulation of pain and it might be involved in the altered pain sensitivity often observed in *SHANK3*-related ASD, due to *SHANK3* role in the modulation of *TRPV1* function and pain transduction^54^. Therefore, we investigated if self-injurious behaviours had been reported for case AB162, and/or if the 2 ASD siblings, discordant for the presence of the disruptive *TRPV1* mutation, could have a different pain tolerance threshold. However, no evidence of altered behavioural responses to pain (tested according to the standard neurological examination) was observed in case AB162, suggesting that *TRPV1* is haplosufficient for the pain-sensitivity phenotype. However, given the involvement of *TRPV1* multiple functions in the nervous system, it is still possible that the *de novo TRPV1* frameshift variant could contribute to modulation of the ASD phenotype.

In conclusion, this study adds relevant data to the large number of CNV studies in autism, all of which are critical for accurate interpretation of results, given the complex contribution of CNVs to ASD etiology. We have also shown that the Illumina PsychArray is a reliable and cost-efficient tool for genic CNVs detection, in addition to providing useful genotyping data, including rare and low-frequency exonic variants previously implicated in psychiatric disorders.

A major limitation in our study is the relatively small sample size, which provides limited power to confidently identify novel rare risk variants for ASD. Therefore, to prioritize variants of interest we relied on previously identified ASD candidate genes. Our data support the contribution of *VPS13B, WWOX, CNTNAP2*, *RBFOX1, MACROD2, APBA2, PARK2*, *GPHN*, and *RNF113A* in ASD susceptibility. Moreover, our study was successful in providing evidence for an enrichment of rare CNVs intersecting SFARI genes and a global increased burden of rare variants in ASD cases compared to controls, thus providing further support for a multigenic model of ASD risk.

## Methods

### Study design

An overview of the experimental design is shown in Fig. 1. Briefly, all DNA samples from 128 ASD individuals, 238 parents and 365 controls were genotyped using the Illumina Infinium® PsychArray microarrays (Illumina, San Diego, California, USA). After quality controls, genotyping data were used to assess the combined contribution of rare CNVs and rare exome variants in this cohort of ASD families. Moreover, we undertook exome sequencing (WES) in the single multiplex family included in this study to test if rare coding mutations might explain the ASD phenotype shared by the two affected brothers in this family.

### Description of sample

A total of 128 Italian individuals with an ASD diagnosis and 238 parents from 127 unique families were recruited at the IRCCS Stella Maris Foundation (Pisa, Italy). All participants were of self-reported Italian ancestry and provided a written informed consent to participate. This sample is independent of the Italian sample included in the Autism Genome Project study^5,27^. See Supplementary Methods for clinical assessment details.

The ASD samples include 106 males and 22 females, with a 4.8:1 male/female ratio. DNA samples from both proband’s parents were available for 111 families, and from a single parent for the remaining ones. All DNA samples were extracted from whole blood.

The main clinical and diagnostic characteristics of all ASD probands are summarized in Table 1.

The control sample consisted of 365 anonymized DNA samples from Italian individuals (M/F:199/166), with no psychiatric disorders (See Supplementary Methods).

### Genotyping and CNV data analysis

All DNA samples from 128 ASD individuals, 238 parents and 365 controls were genotyped using the Illumina Infinium® PsychArray microarrays (Illumina, San Diego, California, USA) in two batches, the first using Illumina PsychArray-24 v1.0, the second using PsychArray-24 v1–1 array.

Genotyping, CNV calling methods and quality control (QC) criteria at both sample-level and CNV call-level are described in Supplementary Methods.

After QC, 729 samples (128 ASD individuals, 238 parents and 363 controls) remained.

Burden analyses for rare CNVs in cases and controls were performed using PLINK v1.07^55^.

To determine whether the observed CNV enrichment is really specific to the subset of ASD candidate genes and not general to all genes, we applied the gene-set enrichment method for CNV data^56^ implemented in the PLINK software package (–cnv-enrichment-test), that is robust to case-control differences in CNV size, CNV rate, and systematic differences in gene size. Given the relatively small sample size, we applied robust permutation testing (*–mperm 10000*) and 1-sided empirical p-values were returned.

As CNV burden analysis is highly susceptible to technical bias, to ensure that CNV ascertainment was consistent among affected individuals and control subjects for which we do not have parental data, we included in the analysis only the CNVs identified in cases before running the “trio option” in PennCNV.

### Burden analysis of single-nucleotide rare variants

Prior to association analysis, additional quality control was performed using PLINK v.1.9^57^. Since the genotyping was carried out in in two batches, we limited our analysis to the 566,178 variants present in both versions of the array. Moreover, in order to deal with differences in SNP names and reference allele, we took advantage of the Illumina PsychArray support files and then we specifically checked: i) SNP allele frequency differences between the two batches, ii) the presence of SNPs with different names but mapping to the same genomic position, iii) SNP strand differences between the two batches using the*–flip-scan* option. Then, SNPs were filtered on missing rate (genotyping missingness > 5%), missing rate differences between cases and controls (*p < *0.001), and deviations from Hardy-Weinberg equilibrium (*p* < 0.001). Post QC, non-monomorphic SNPs were mapped to RefSeq genes and association analysis was performed on rare genic variants. For frequency filtering we retained only variants with a MAF ≤ 0.05 in our dataset of 748 samples (128 ASD individuals, 238 parents and 382 controls) and a MAF ≤ 0.01 in gnomeAD exome, gnomeAD genome (https://gnomad.broadinstitute.org/) and the 1000 Genomes Project (http://www.internationalgenome.org/).

Finally, we generated a final subset of SNPs pruned for linkage disequilibrium (200 kb window size, r^2^ > 0.5), in order to avoid inflation of type I error due to presence of intermarker LD.

Rare variant global burden association analysis was performed using the Burden of Rare Variants Transmission Disequilibrium Test (TDT-BRV), an extension of the TDT. The TDT-BRV method counts the number of minor-allele-transmitted events and major-allele-transmitted events from every informative parent to the affected proband^24^.

### Ethical approval and informed consent

All participants provided a written informed consent to participate to this study. This study was approved by the local Ethical Committee (Fondazione Stella Maris, IRCCS; protocol number 05/2011). All research was performed in accordance with the relevant guidelines and regulations.

## Supplementary information

Supplementary information

Supplementary information 2

## Data Availability

All data generated or analysed during this study are included in this published article and its Supplementary Information files.
