# Supplementary material for: An integrated analysis of rare CNV and exome variation in Autism Spectrum Disorder using the Infinium PsychArray
Source: Sci Rep. 2020 Feb 21;10:3198. doi: 10.1038/s41598-020-59922-3 (PMC7035424; doi:10.1038/s41598-020-59922-3)
Supplement: Supplementary file 1 — Supplementary information [file 41598_2020_59922_MOESM1_ESM.docx]

**An integrated analysis of rare CNV and exome variation in Autism Spectrum**

**Disorder using the Infinium PsychArray**

**(Supplementary Material)**

Elena Bacchelli^1^*, Cinzia Cameli^1+^, Marta Viggiano^1+^, Roberta Igliozzi^2^, Alice Mancini^2^, Raffaella Tancredi^2^, Agatino Battaglia^2^, Elena Maestrini^1^*

^1^ Department of Pharmacy and Biotechnology, University of Bologna, Bologna, Italy

^2^ IRCCS Stella Maris Foundation, Viale del Tirreno 331, 56128, Calambrone, Pisa, Italy.

* Corresponding authors

E-mail: [elena.maestrini@unibo.it](mailto:elena.maestrini@unibo.it) (EM)

E-mail: [elena.bacchelli@unibo.it](mailto:elena.bacchelli@unibo.it) (EB)

^+^These authors contributed equally to this work.

**Supplementary Methods**

**Clinical assessment and description of samples**

A detailed morphological clinical evaluation was performed by one of the authors (A.B.) for all ASD subjects in order to exclude known genetic syndromes associated with autism. Fragile-X testing, array-based comparative genomic hybridization (aCGH), EEG, and neurometabolic work-up, were obtained for all probands. Brain MRI was performed when clinically appropriate.

ASD diagnosis was usually performed according to the “gold standard” diagnostic tools ADI-R and ADOS (*1-3*). In particular 95 individuals met criteria on one (either ADI-R or ADOS-G/ADOS-2), and 39 on both diagnostic tools. The remaining 33 received an ASD diagnosis on clinical grounds by an expert child psychiatrist (R.T), according to the DSM-IV or DSM-5 (*4, 5*).

Adaptive functioning was assessed in 108 ASD individuals using the Vineland Adaptive Behavior Scale (VABS)(*6*). 106/128 individuals were assessed with standardized measures of cognitive level (IQ) (*7-12*) or of developmental level (DQ) (*13, 14*). The Child Behavior Check-List (CBCL)(*15, 16*) was administered to the parents of 97 propositi to investigate the presence/absence of associated internalizing/externalizing problems.

The control sample consisted of 365 Italian individuals without psychiatric history, recruited at the Division of Toxicology and Clinical Pharmacology, Headache Centre, University of Modena and Reggio Emilia (Italy), as part of a study on the genetics of nicotine dependence (*17*), following the approval by the Human Research Ethics Committee of Modena (protocol number 2224/2013).

**CNV data Analysis**

The clustering algorithm implemented in GenomeStudio was used to cluster the data. GenomeStudio was also used to evaluate all genotypes using a quantitative genotype quality score called GenCall (GC) score, ranging from 0 to 1 with 1 being the best. The quality score cutoff was set at 0.15.

Quality control was first performed based on the whole-genome genotyping data before CNV calling. Samples were required to have a minimum call rate of 0.95. SNPs quality control (QC) was performed according to recommended guidelines (*18*).

CNV calls were made using three different CNV detection algorithms: PennCNV (*19*), QuantiSNP (*20*) and CNVPartition (Illumina). For CNV analysis using PennCNV, we used default HMM parameters, while we built a custom population B allele frequency (PBF) file, where the PFB values for all SNPs have been compiled from 960 samples using the “compile_pfb.pl” script in PennCNV. SNP with a PFB value of 0 or 1 have been treated as non-polymorphic markers (or as intensity-only markers) by setting its PFB to 2. We also generate a GC-model signal adjustment file specific for the PsychArray using the script “cal_gc_snp.pl”, in order to apply the adjustment for genomic waves, before CNV calling to reduce false positive calls.

We removed samples that were outliers with respect to LRR (log R ratio), BAF (B allele frequency) and an excessive number of CNVs detected. Specifically, the CNV number and the standard deviations (s.d.) for the LRR and BAF for an individual sample were required to be within the mean±three times the s.d. for the entire cohort for each of these parameters. According to these criteria, we excluded 2 controls from further analysis.

All CNVs went through QC filtering. Firstly, raw CNVs in the same sample were joined together if the distance between them was< 50% of their combined length. For inclusion, CNVs required calling by at least two algorithms (with one being PennCNV) with moderately high confidence (confidence criteria >15) and overlapped by at least 50%. If the CNV boundaries varied between the different calling algorithms, we retained the largest one. Finally, the final set of stringent CNVs were selected according to these criteria: less than 50% of their length overlapping segmental duplications, ≥10 kb in size, and detected by at least 5 probes.

PennCNV, and specifically its trio option, was used to confirm inheritance status of the resulting CNV calls. We performed manual visual review of the BAF and LRR plots of the significant CNV loci identified.

**Validation of CNV calls**

We selected a subset of CNVs for validation (see Supplementary Table S1) by quantitative PCR (qPCR) using SsoAdvanced™ Universal SYBR® Green Supermix (BIORAD). These included three categories: (i) 5 putative genic *de novo* CNVs, (ii) 11 CNVs deemed relevant (overlapping known genomic disorders loci and/or intersecting ASD candidate genes), (iii) 15 CNVs identified by a number of probes between 5 and 10 or being between 10 and 15 kb in length. Each assay was conducted in triplicate, using 25 ng of genomic DNA in each reaction and with at least three sets of primers corresponding to the region of interest and another mapping to a control region on FOXP2 gene. Moreover, the edges of all clinically relevant CNVs were confirmed by designing qPCR probes in regions between the minimum and maximum CNV boundaries as predicted by calling algorithms. The parents were also tested to confirm CNV inheritance. Comparative Ct method was used in order to calculate the fold change of copy number. qPCR data are shown in Supplementary Figure S1.

**CNV classification criteria**

Stringent CNVs that passed all QC filters (≥5 probes, ≥10 kb size) were classified considering their type (gain and loss), size, location, gene content and inheritance pattern.

To assess the clinical relevance of CNVs, we first identified all exonic CNVs that intersect loci of micro-deletion/duplication syndromes listed in the DECIPHER database (<https://decipher.sanger.ac.uk/>)(*21*), and recurrent CNV shown to increase the risk of developing early-onset neurodevelopmental disorders (developmental delay, autism spectrum disorders, and various congenital malformation)(*22*).

Then, to define rare CNVs, we first selected all CNVs with an overlap >75% with a copy number stable regions of the genome, according to the stringent CNV map of the human genome (*23*). Next, we identified rare variants as those present in ≤1% of our sample set of 601 subjects (238 ASD parents and 363 controls, all genotyped on the same array), using the 50% reciprocal overlap criteria (*24*).

**ASD candidate genes**

To define ASD candidate genes we used the SFARI gene database (<https://gene.sfari.org/>, release July 2018) and its ranking system (https://gene.sfari.org/about-gene-scoring/), including SFARI genes for syndromic disorders that have ASD as part of the phenotype (Category S), genes that are identified as having the strongest evidence for involvement based on rigorous statistical case-control comparisons (Categories 1 and 2) and genes with a suggestive or minimal evidence of involvement using either common or rare variant approaches (Categories 3 and 4). The complete list of ASD genes used in this study and referred as “SFARI gene-set” is provided in Supplementary Table S4.

**Expression analysis**

Total RNA from proband AB151 and his mother, and 2 healthy control subjects was extracted from whole blood stored in RNAlater using the RiboPure™-Blood Kit (Life Technologies) and quantified by NanoDrop. cDNA was synthesized using the SuperScript™ III First-Strand Synthesis SuperMix for qRT-PCR (Invitrogen) using approximately 400 ng of RNA as template as template. Then, 20 ng of cDNA were used to test *VPS13B* expression by quantitative reverse transcription PCR (qRT-PCR) with SYBR Green. We used two different primers pairs covering exons 5–6, common to all *VPS13B* isoforms, and exons 23–24, specific to the two full-length isoforms, that are disrupted by the deletion in case AB151. Quantification of the expression level of VPS13B was performed in comparison the housekeeping gene *TFRC* as a reference gene.

**Exome sequencing**

We undertook exome sequencing (WES) in the single multiplex family included in this study (AB162 & AB163). Genomic DNA from AB162, AB163 and their unaffected parents was enriched for exonic sequences using the Nextera exome enrichment kit (Illumina Inc., San Diego, CA, USA) and sequenced as 100-bp paired-end reads on the Illumina HiSeq2500 platform (Illumina Inc., San Diego, CA, USA). We required that each exomes had a read depth (DP) of 10X or more for 90% of the total exome coverage and 20X or more for 80%. WES data analysis was performed using CoVacS (*25*), a recently developed pipeline that uses a consensus calling approach based on three different algorithm (GATK, Varscan and Freebayes). VCF files generated by CoVaCS were annotated using Annovar (*26*). The gene-based annotation (position, nomenclature, gene name, gene function) was performed using RefSeq. In order to remove low-quality variants called genotypes were required to have DP ≥ 10, and Genome Quality (GQ) ≥ 20. We selected only rare variants with MAF ≤ 1% in gnomeAD exome, gnomeAD genome (http://gnomad.broadinstitute.org/) and the 1000 Genomes Project (http://www.1000genomes.org/). We considered in our analysis only Likely Gene Disrupting (LGD) and damaging missense mutations. LGD mutations included stop-gain, stop-loss, frameshift and splicing mutations, while damaging missense mutations were defined as deleterious by at least two of the following criteria: SIFT score ≤ 0.05, Polyphen2 (HDIV) score ≥ 0.95, Mutation Assessor ≥ 2, Phred transformed CADD score ≥ 15, placental mammal PhyloP ≥ 2.4, vertebrate PhyloP ≥ 4 (*27*).

**Supplementary tables**

**Table S1. CNVs validated by qPCR.**

File name: **Supplementary_Tables.xlsx, sheet “Table S1”** (Excel file .xlsx)

**Table S2. Size-wise comparison of rare genic CNV calls made by the Illlumina 1M-duo array versus the infinium PsychArray in an ASD individual with a 15q13.3 duplication**

File name: **Supplementary_Tables.xlsx, sheet “Table S2”** (Excel file .xlsx)

**Table S3. List of rare CNVs (<1% frequency) identified in 128 ASD cases and 363 controls, used for burden analysis**

File name: **Supplementary_Tables.xlsx, sheet “Table S3”** (Excel file .xlsx)

**Table S4. SFARI gene set**

File name: **Supplementary_Tables.xlsx, sheet “Table S4”** (Excel file .xlsx)

**Table S5: Inherited CNVs from 23 probands, intersecting ASD candidate genes.**

File name: **Supplementary_Tables.xlsx, sheet “Table S5”** (Excel file .xlsx)

*CNVs highlighted in bold are discussed in the main text; ^a^ Frequency in 363 controls (C) and 238 parents (P): TP: transmitting parent; NP: non-transmitting parent; ^b^ PDD: pervasive developmental disorder according to the DSM-IV; PDD-NOS: pervasive developmental disorder not otherwise specified; ASD: autism spectrum disorder according to the DSM-5; ID: intellectual disability; BCI: borderline cognitive impairment.*

**Table S6. Prioritized rare variants identified by WES of the multiplex family** **AB162/AB163**

File name: **Supplementary_Tables.xlsx, sheet “Table S6”** (Excel file .xlsx)

*Variant validated by Sanger sequencing are highlighted in bold; ^#^pLI score ≥0.9 indicating genes strongly intolerant to loss of function variants are underlined*

**Supplementary Figures**

**Supplementary Figure S1. CNVs validated by qPCR**

*For each of the 31 validated CNVs, panel a) represents the UCSC Table Browser (GRCh37/hg19) screenshot of the genomic region including the CNV (deletions in red and duplication in blue) and panel b) reports the qPCR validation results*

**Supplementary references**

1. Rutter, M., Le Couteur, A. & Lord, C. (Los Angeles, CA: Western Psychological Services, 2003).

2. Lord, C. *et al.* The autism diagnostic observation schedule-generic: a standard measure of social and communication deficits associated with the spectrum of autism. *J Autism Dev Disord* **30**, 205-223 (2000).

3. Lord, C. *et al.* *Autism Diagnostic Observation Schedule, Second Edition*. (Torrance, CA: Western Psychological Services, 2012).

4. *Diagnostic and statistical manual of mental disorders (4th ed.)*. (American Psychiatric Association.  Washington DC: American Psychiatric Association, 1994).

5. *Diagnostic and Statistical Manual of Mental Disorders, Fifth Edition (DSM-5)*. (American Psychiatric Association. Washington, DC: American Psychiatric Association, 2013).

6. Sparrow, S. S., Balla, D. A. & Cicchetti, D. V. (Circle Pines, MN: American Guidance Service. , 1984).

7. Roid, G. H. & Miller, L. J. *Leiter-R*. (Firenze, Italy: Giunti O.S., 1997).

8. Wechsler, D. *Wechsler intelligence scale for children—revised.*, (New York: Psychological Corporation, 1974).

9. Wechsler, D. *Wechsler Intelligence Scale for Children (3rd ed.)*. (New York: Psychological Corporation, 1991).

10. Wechsler, D. *The Wechsler Preschool and Primary Scale of Intelligence – WPPSI-III Technical and Intrepretive Manual 3rd edn*. (Psychological Corporation: San Antonio, TX, USA, 2002).

11. Wechsler, D. *Wechsler Intelligence Scale for Children-WISC-IV*. (Psychological Corporation: San Antonio, TX, USA, 2003).

12. Kaufman, A. S. & Kaufman, N. L. *Kaufmann Brief Intelligence Test, 2nd Edn.*, (Bloomington, MN: Pearson Assessments, 2004).

13. Griffiths, R. (Firenze, IT: Giunti O.S., 2007).

14. Luiz, D. *et al.* *GMDS-ER 2-8 – Griffiths Mental Development Scales Extended Revised: 2 to 8 years (Cianchetti, C., & Sannio Fancello G. trad.)*. (Firenze, IT: Giunti O.S., 2007).

15. Achenbach, T. M. & Rescorla, L. A. (Burlington, VT: University of Vermont, Research Center for Children, Youth & Families.   , 2000).

16. Achenbach, T. M. & Rescorla, L. A. *Manual for the ASEBA School-Age Forms & Profiles.* (Burlington, VT: University of Vermont, Research Center for Children, Youth & Families., 2001).

17. Cameli, C.  *et al.* Genetic variation in CHRNA7 and CHRFAM7A is associated with nicotine dependence and response to varenicline treatment. *Eur J Hum Genet* **26**, 1824-1831 (2018).

18. Guo, Y. *et al.* Illumina human exome genotyping array clustering and quality control. *Nat Protoc* **9**, 2643-2662 (2014).

19. Wang, K. *et al.* PennCNV: an integrated hidden Markov model designed for high-resolution copy number variation detection in whole-genome SNP genotyping data. *Genome Res* **17**, 1665-1674 (2007).

20. Colella, S. *et al.* QuantiSNP: an Objective Bayes Hidden-Markov Model to detect and accurately map copy number variation using SNP genotyping data. *Nucleic Acids Res* **35**, 2013-2025 (2007).

21. Firth, H. V. *et al.* DECIPHER: Database of Chromosomal Imbalance and Phenotype in Humans Using Ensembl Resources. *Am J Hum Genet* **84**, 524-533 (2009).

22. Kirov, G. *et al.* The penetrance of copy number variations for schizophrenia and developmental delay. *Biol Psychiatry* **75**, 378-385 (2014).

23. Zarrei, M., MacDonald, J. R., Merico, D. & Scherer, S. W. A copy number variation map of the human genome. *Nat Rev Genet* **16**, 172-183 (2015).

24. Pinto, D. *et al.* Functional impact of global rare copy number variation in autism spectrum disorders. *Nature* **466**, 368-372 (2010).

25. Chiara, M. *et al.* CoVaCS: a consensus variant calling system. *BMC Genomics* **19**, 120 (2018).

26. Wang, K., Li, M. & Hakonarson, H. ANNOVAR: functional annotation of genetic variants from high-throughput sequencing data. *Nucleic Acids Res* **38**, e164 (2010).

27. Yuen, R. K. *et al.* Genome-wide characteristics of de novo mutations in autism. *NPJ Genom Med* **1**, 160271-1602710 (2016).


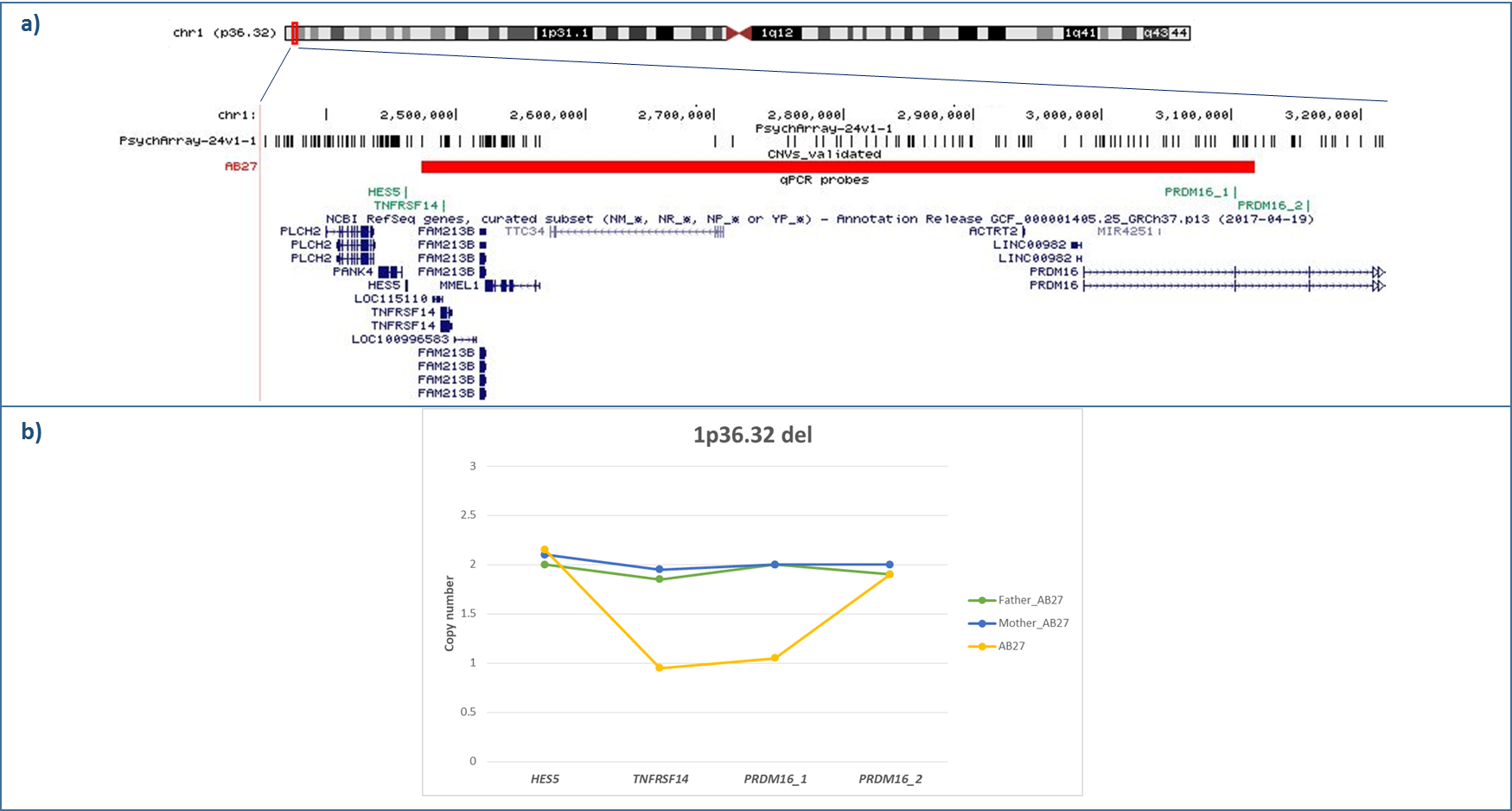
**Supplementary Figure S1_1**

**Supplementary Figure S1_2**


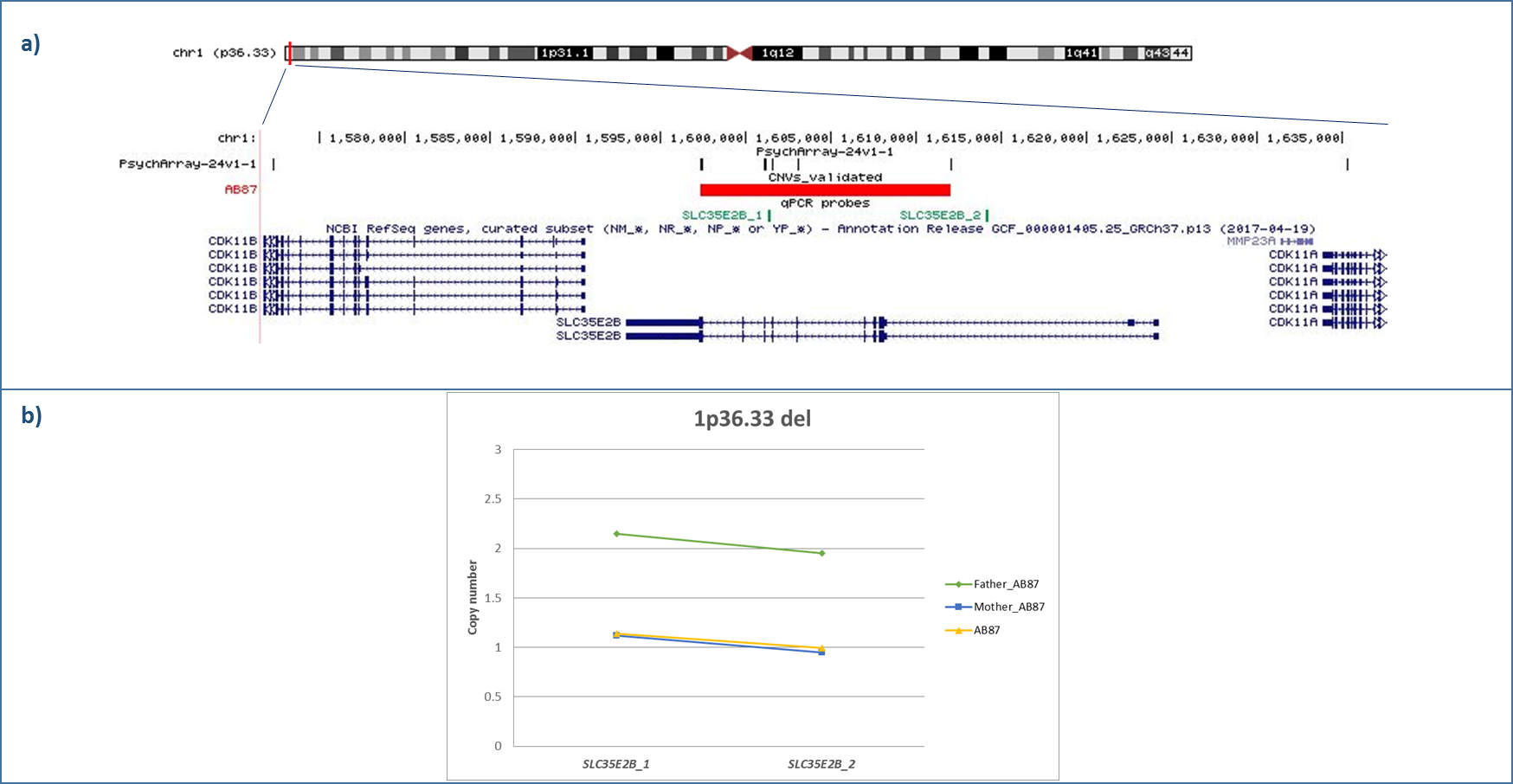


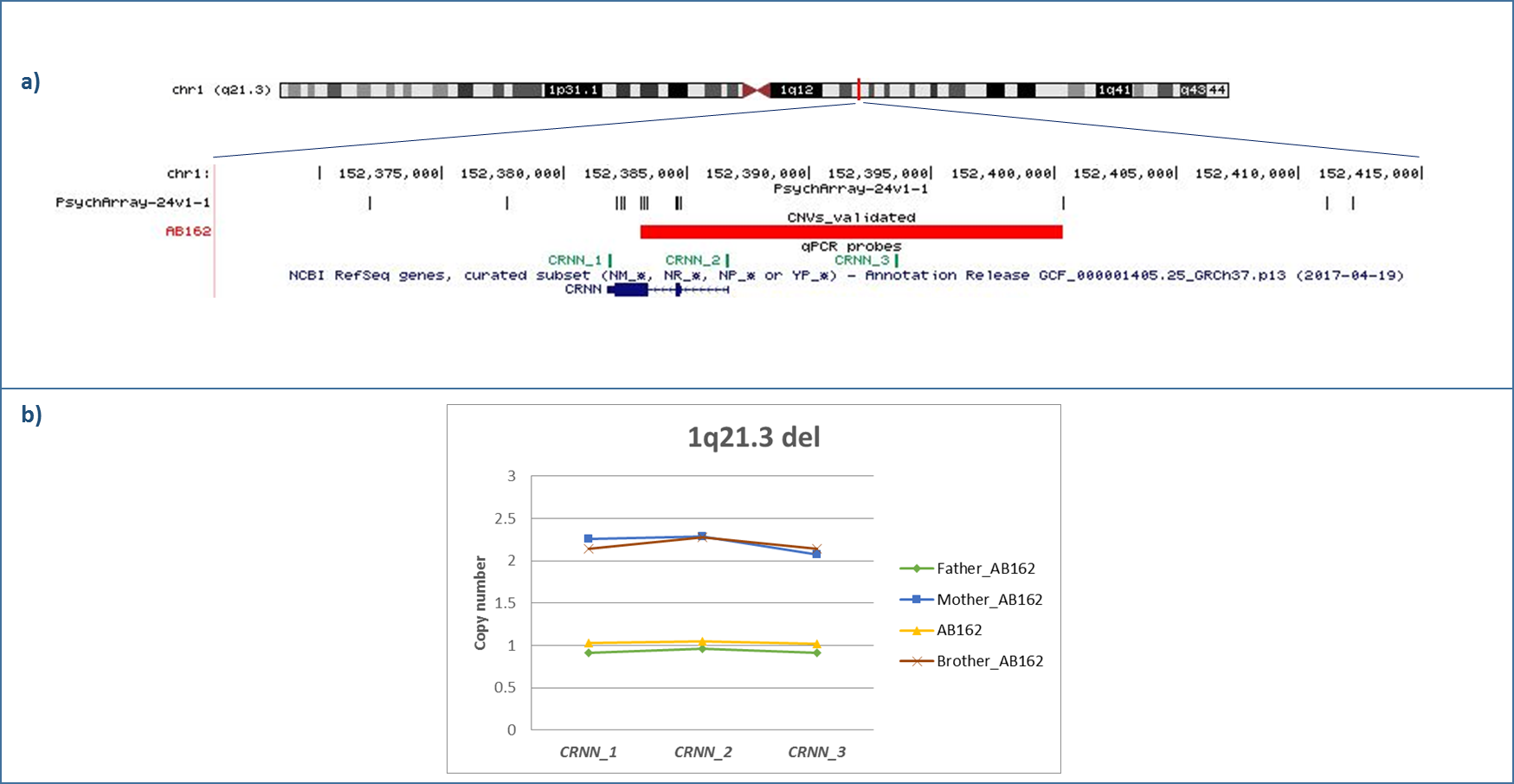
**Supplementary Figure S1_3**


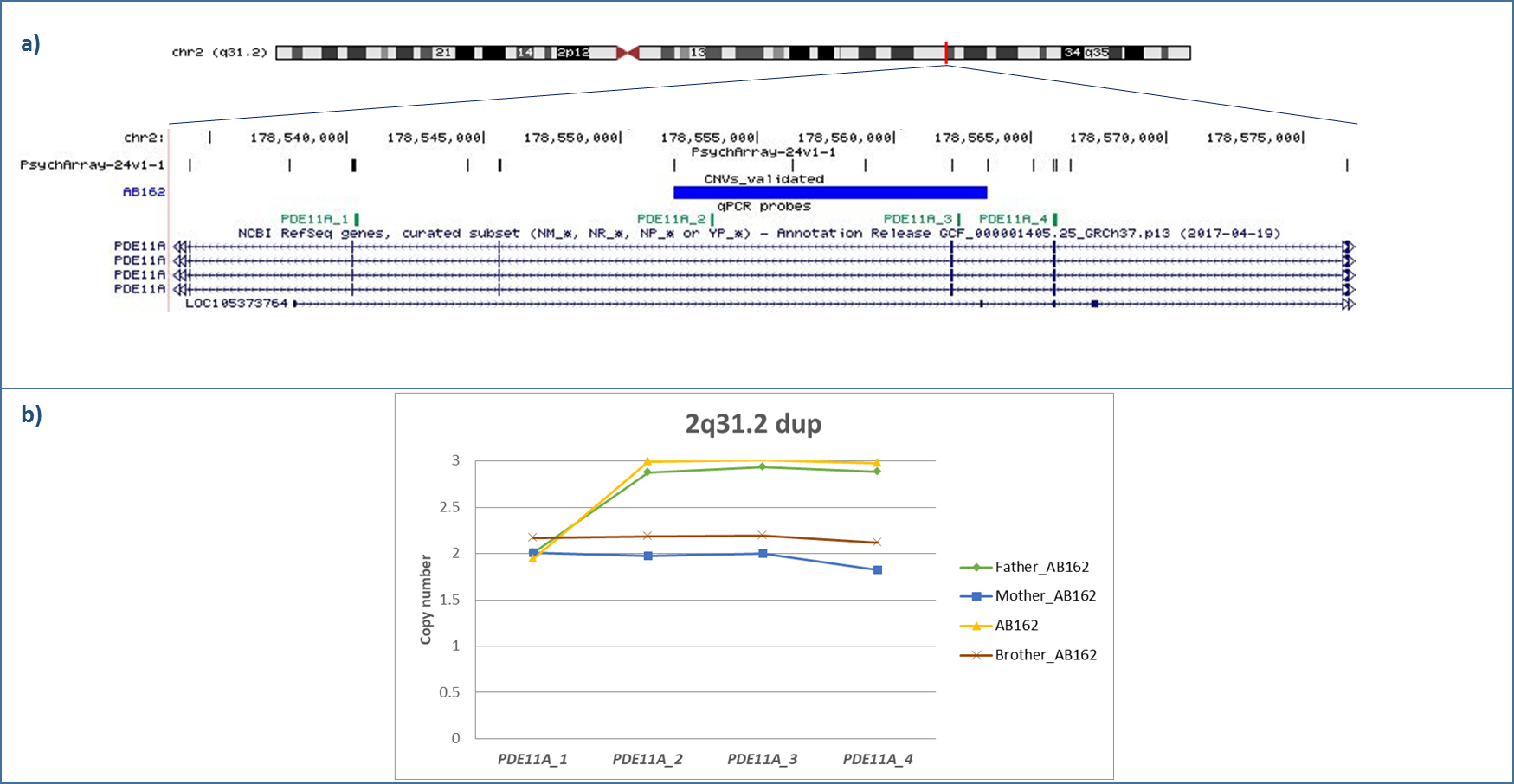
**Supplementary Figure S1_4**

**Supplementary Figure S1_5**


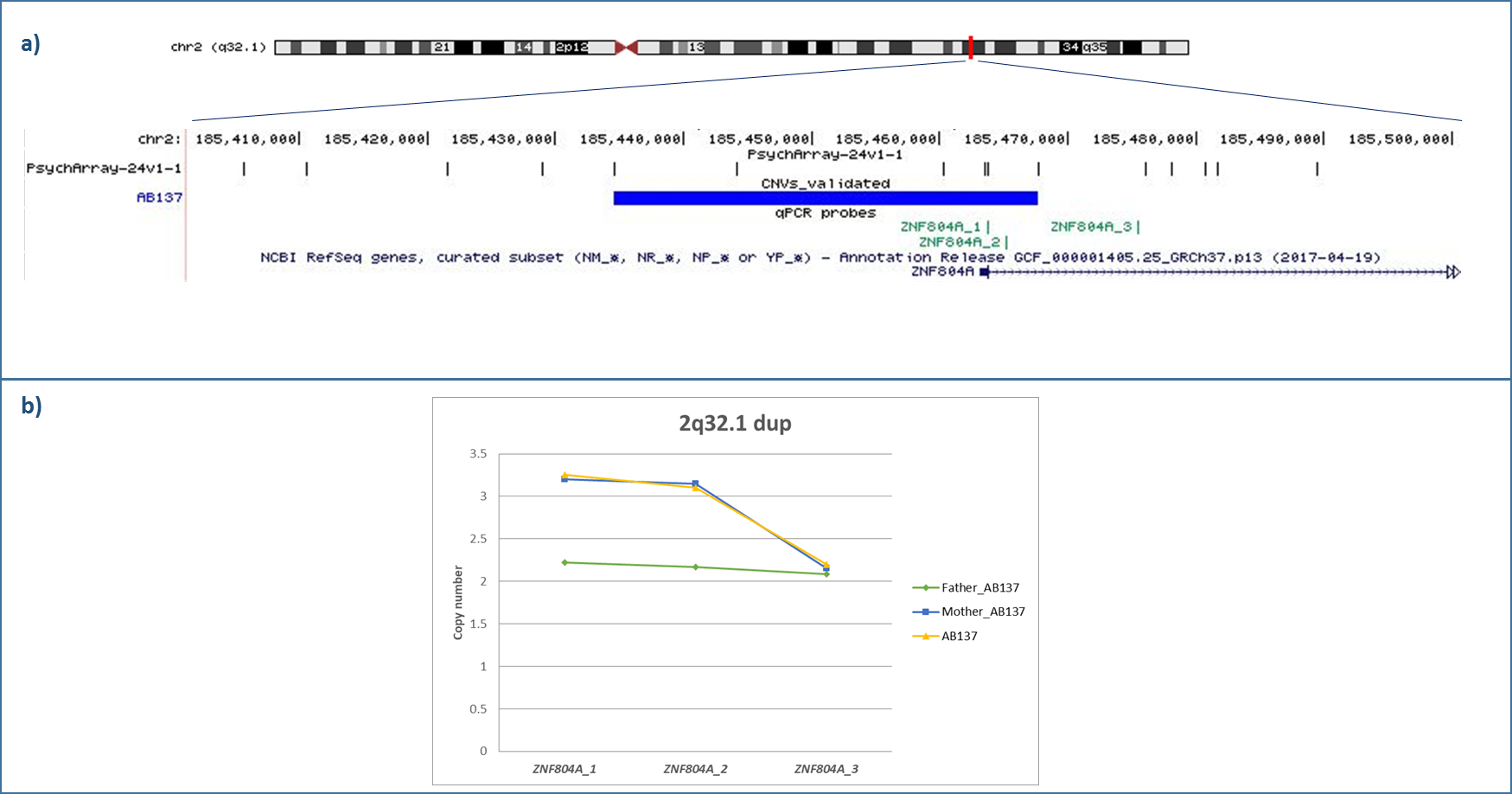


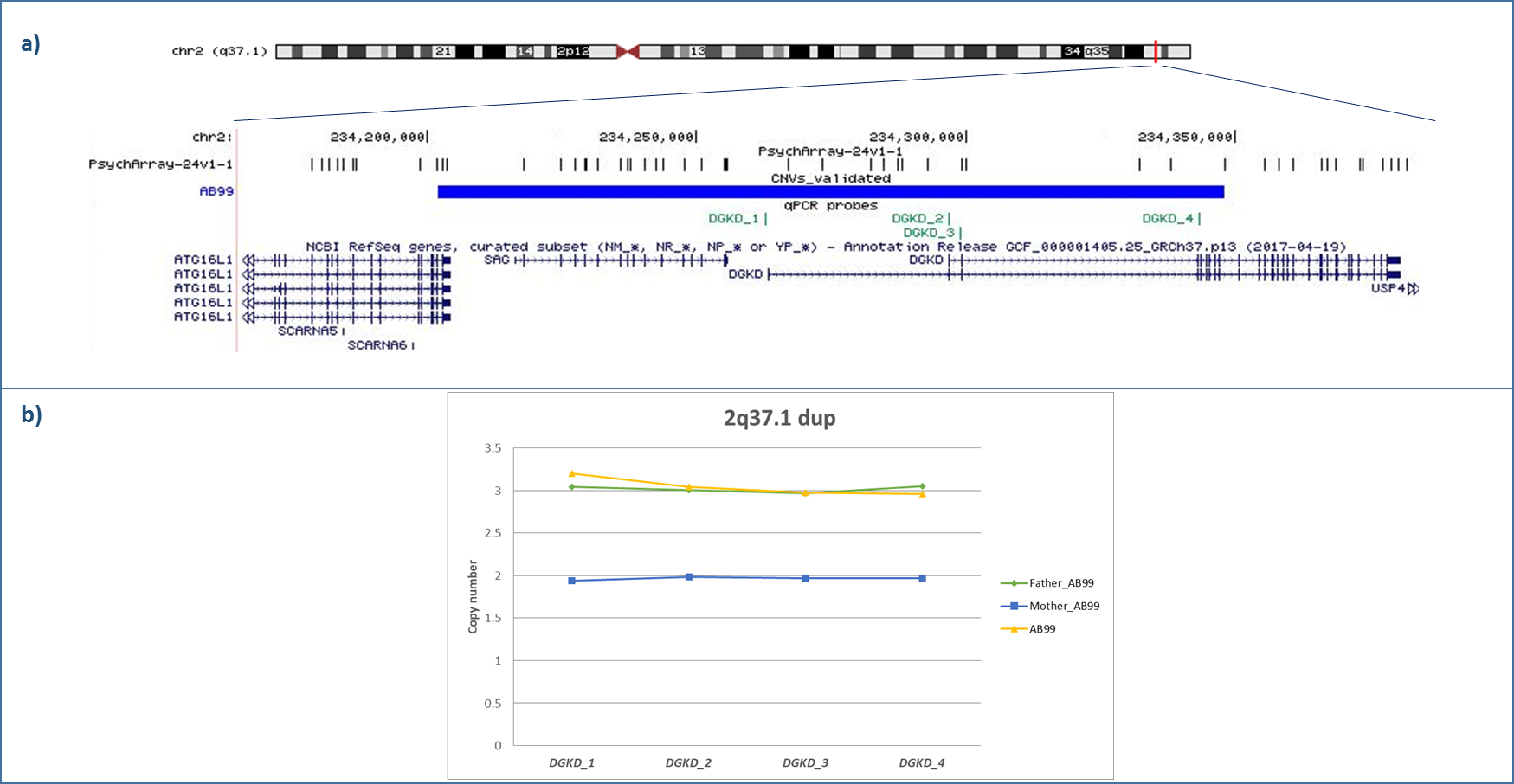
**Supplementary Figure S1_6**


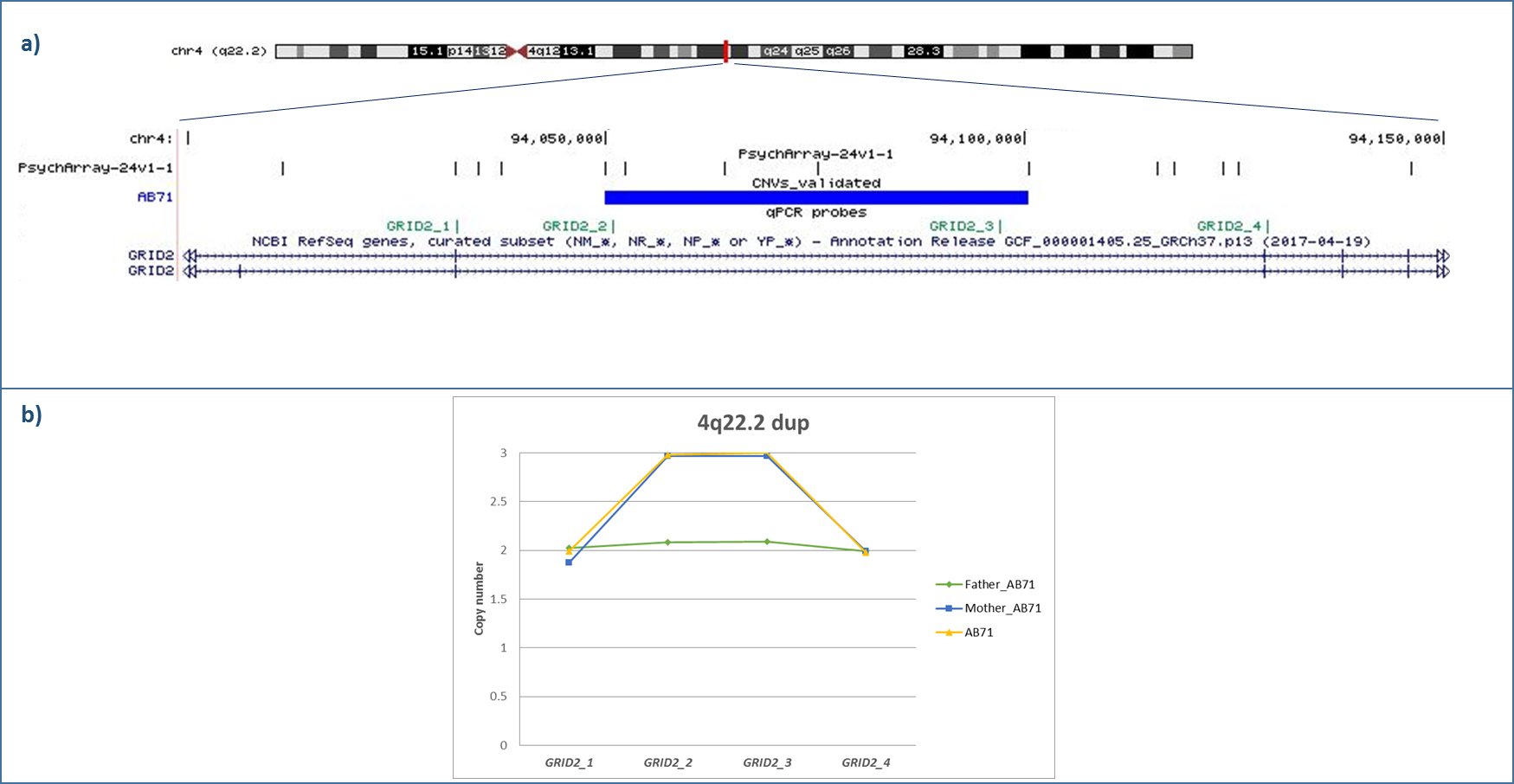
**Supplementary Figure S1_7**

**Supplementary Figure S1_8**


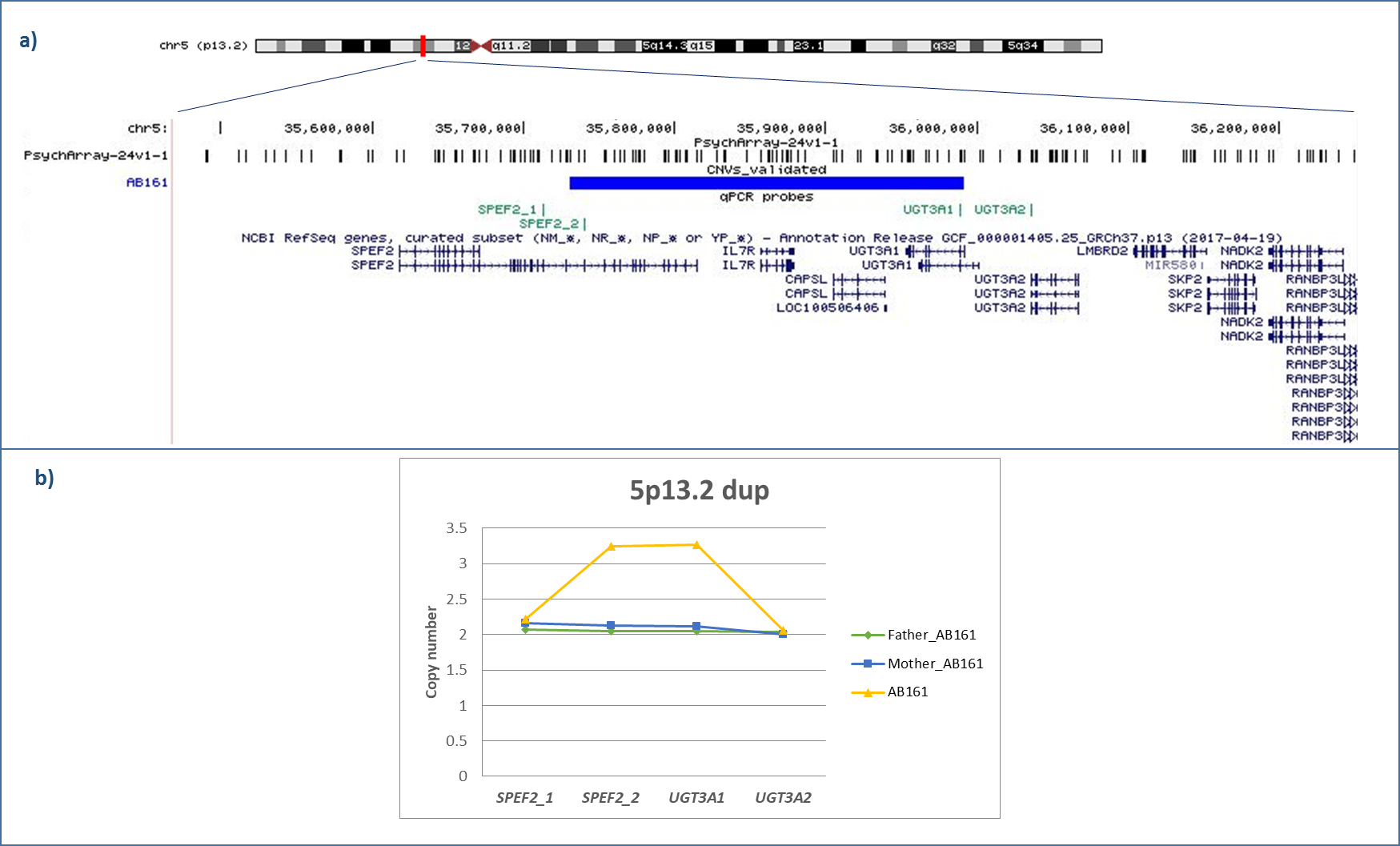


**Supplementary Figure S1_9**


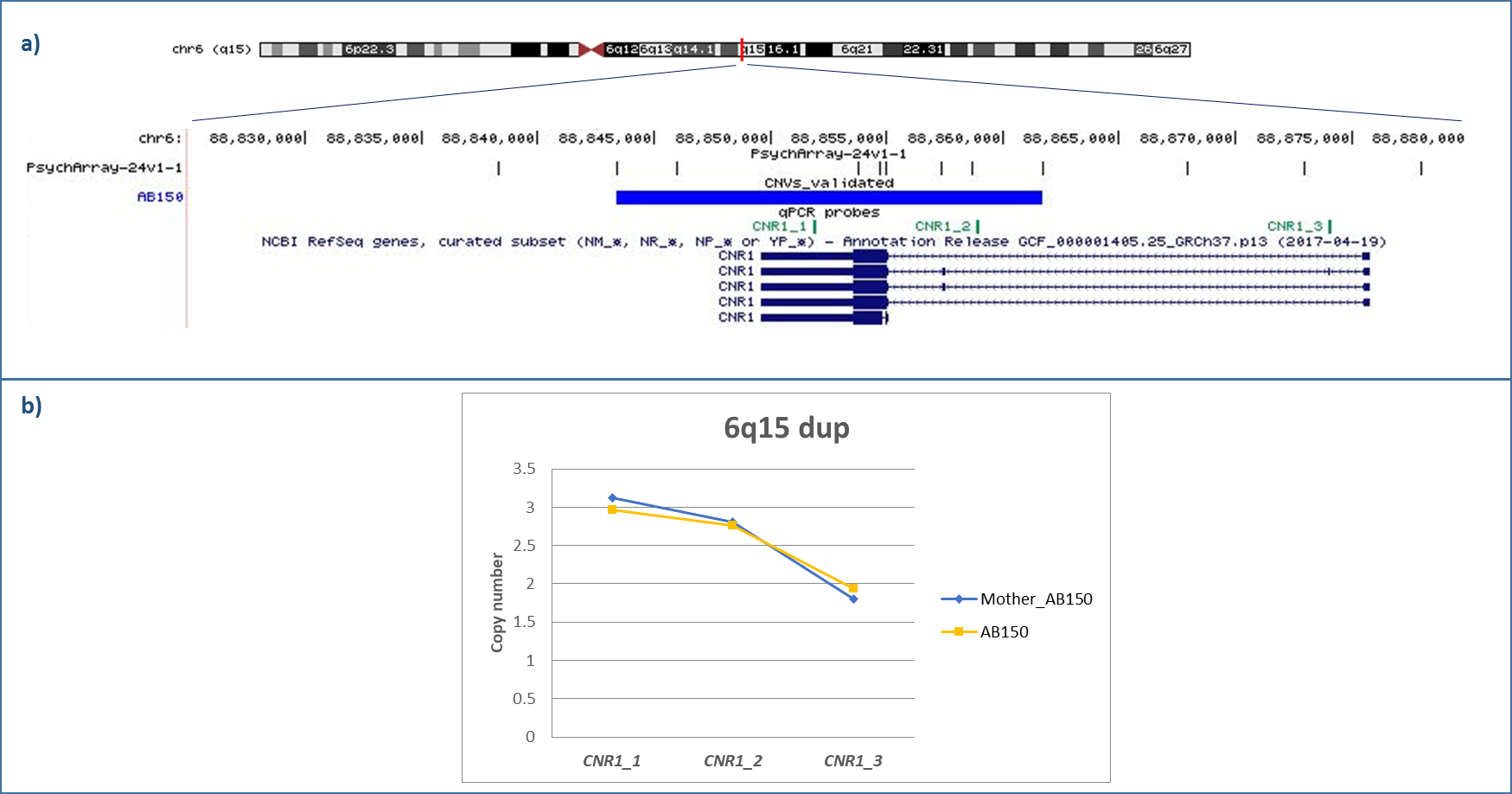


**Supplementary Figure S1_10**


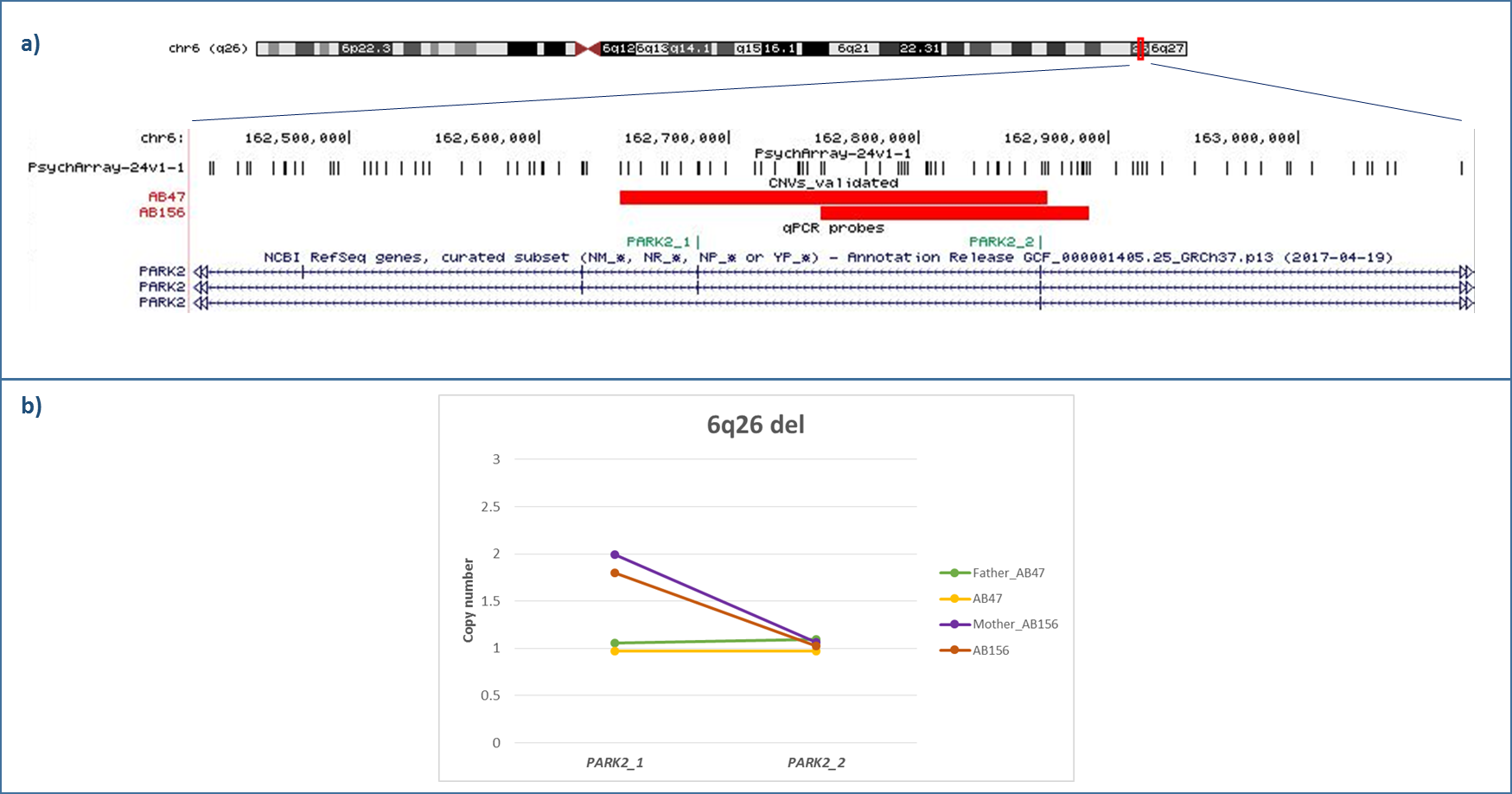


**Supplementary Figure S1_11**


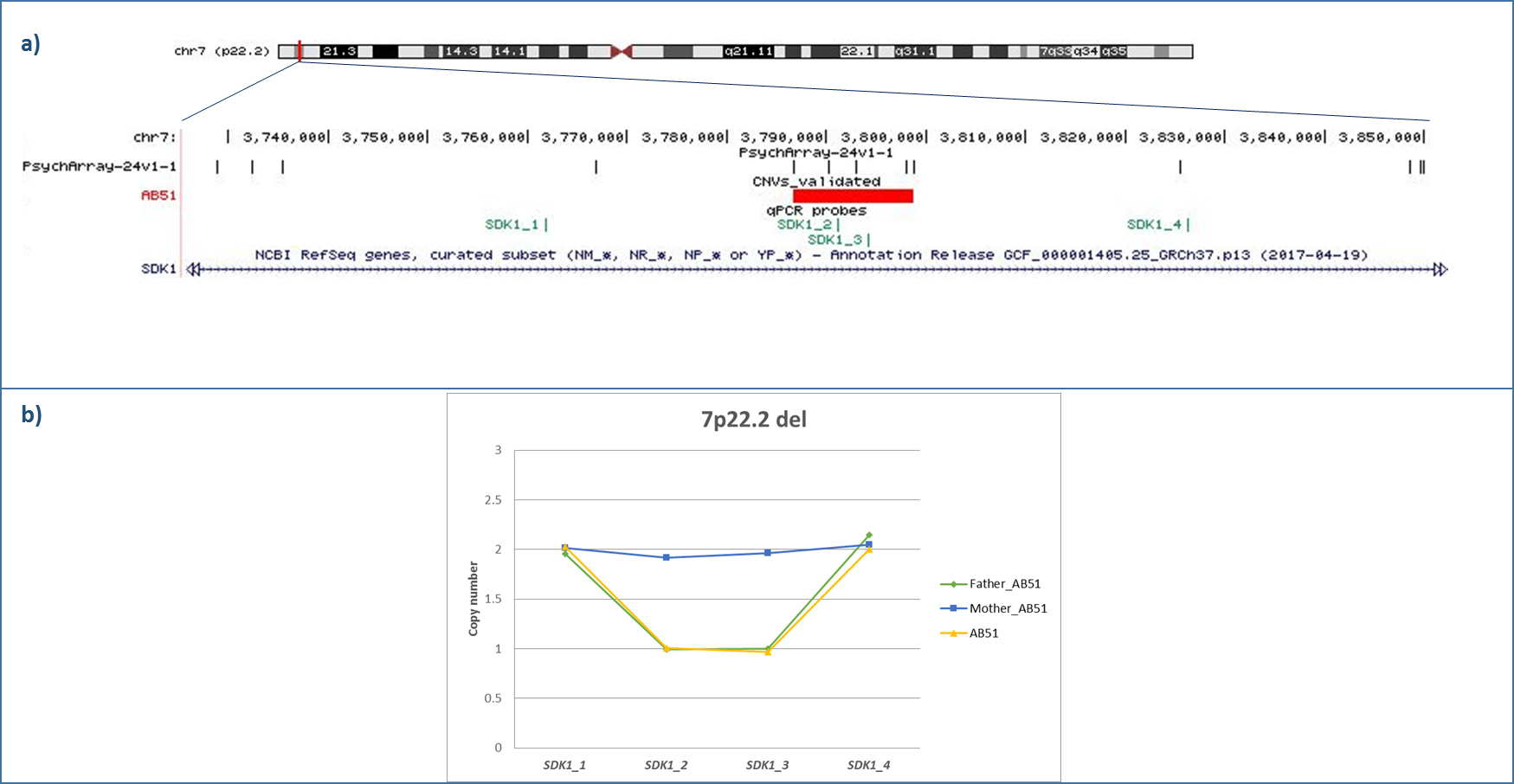


**Supplementary Figure S1_12**


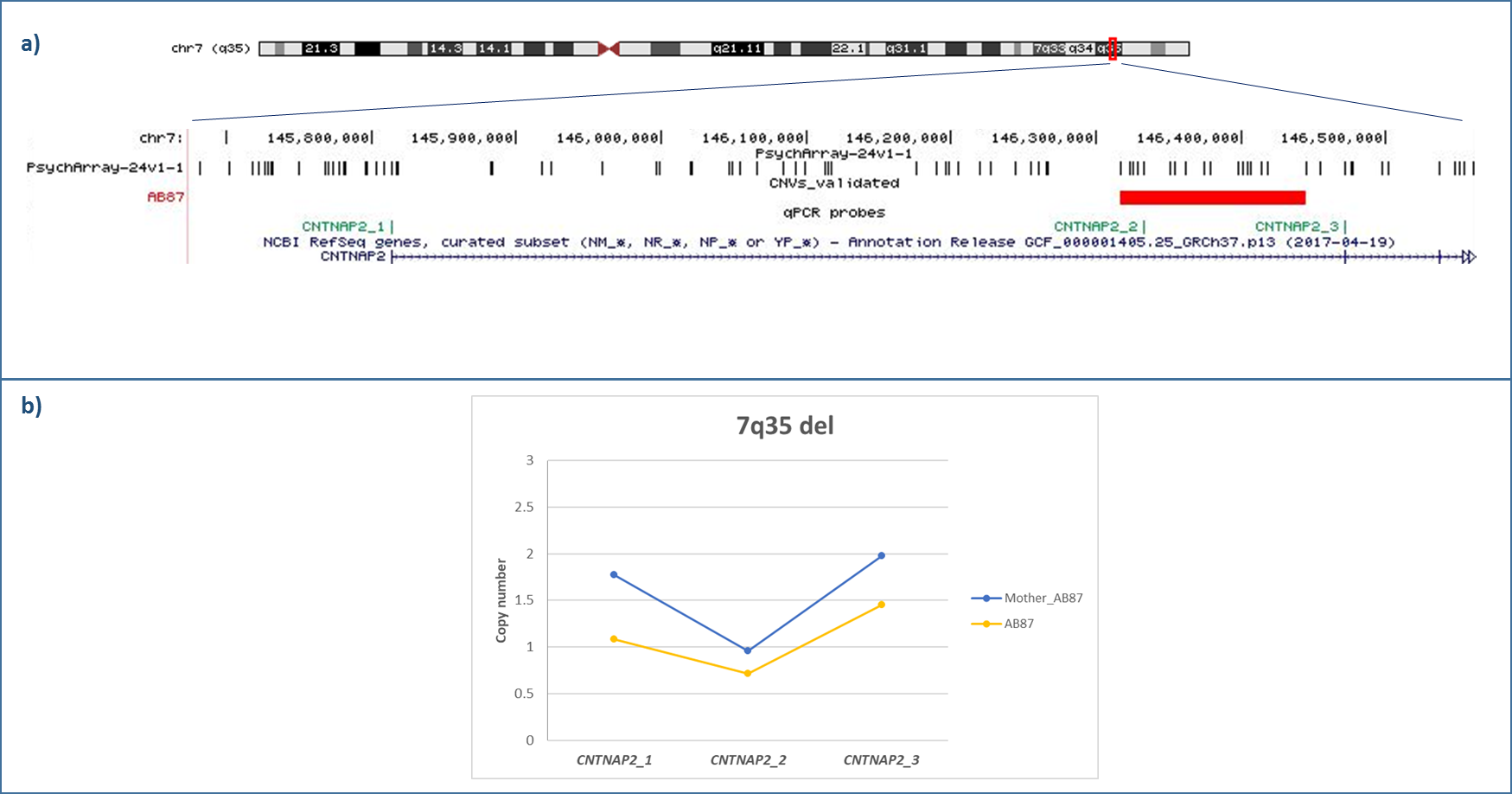


**Supplementary Figure S1_13**


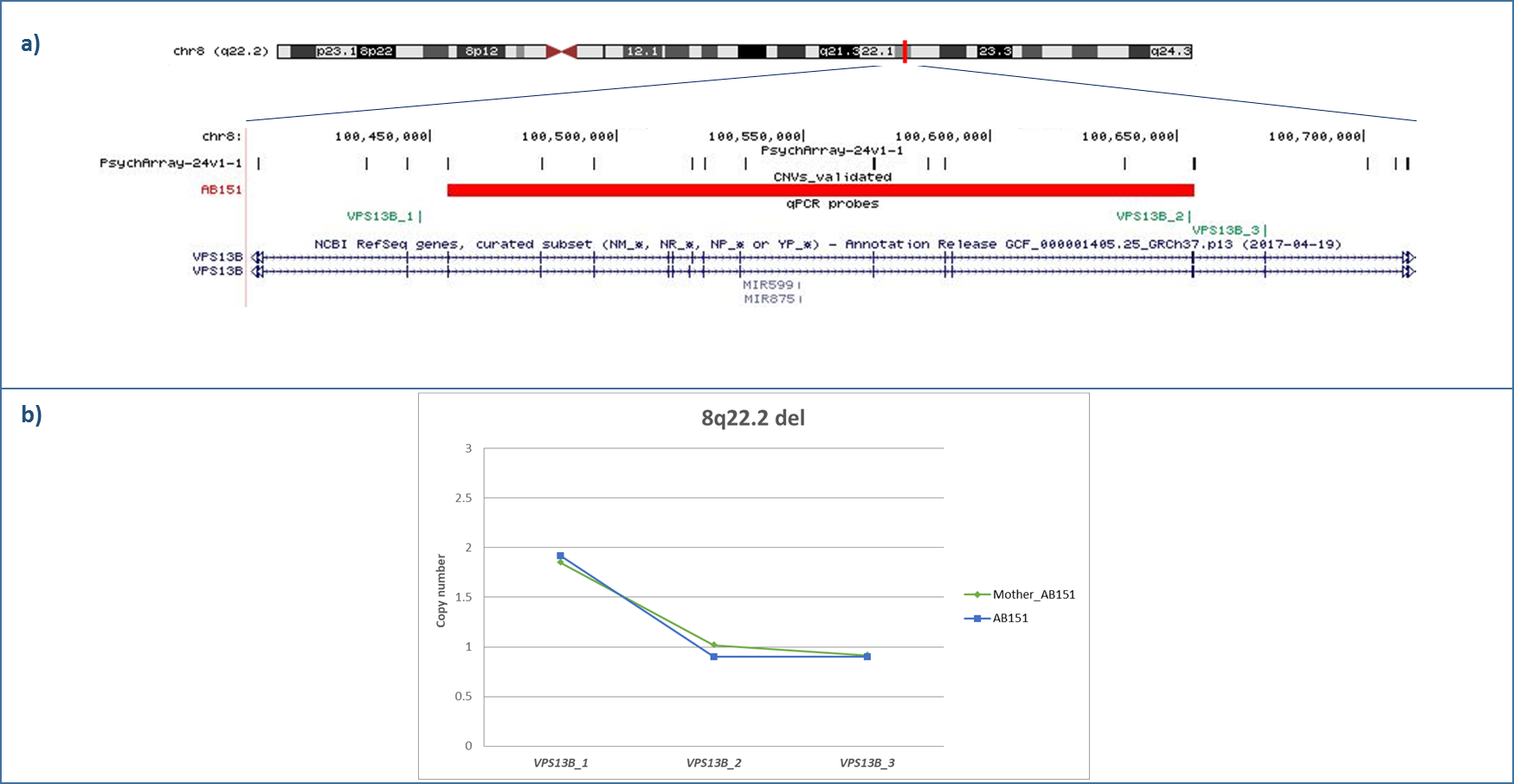


**Supplementary Figure S1_14**


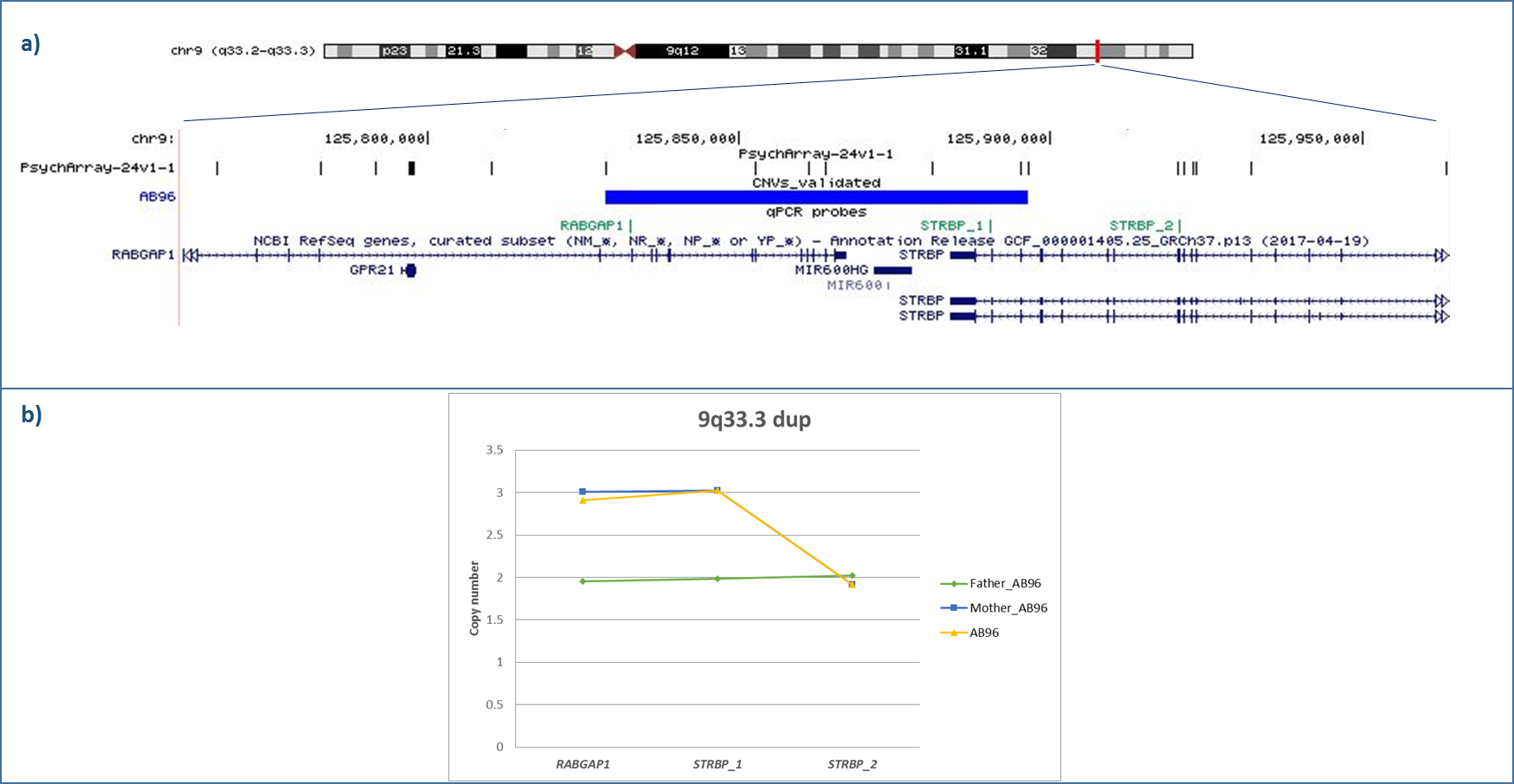


**Supplementary Figure S1_15**


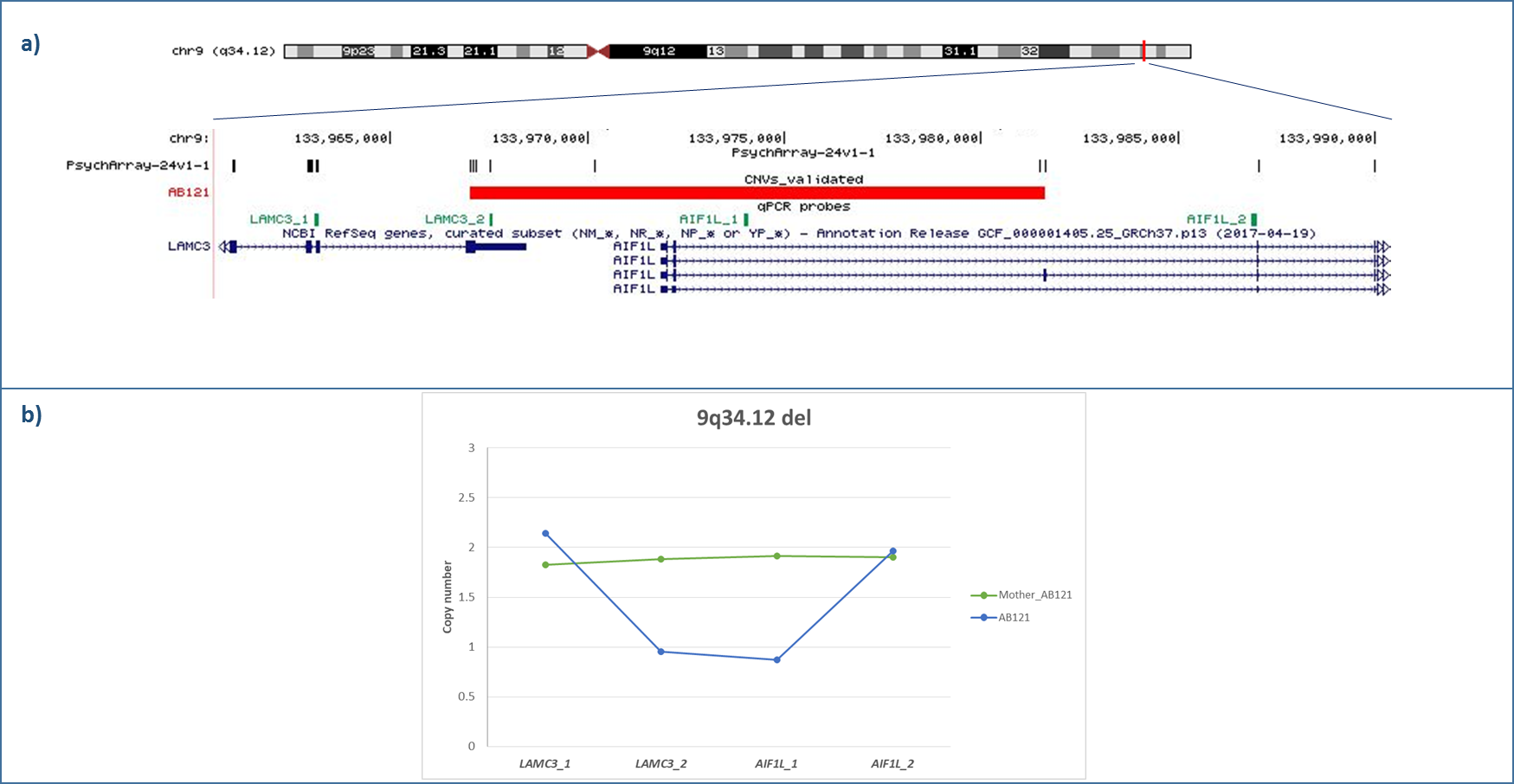


**Supplementary Figure S1_16**


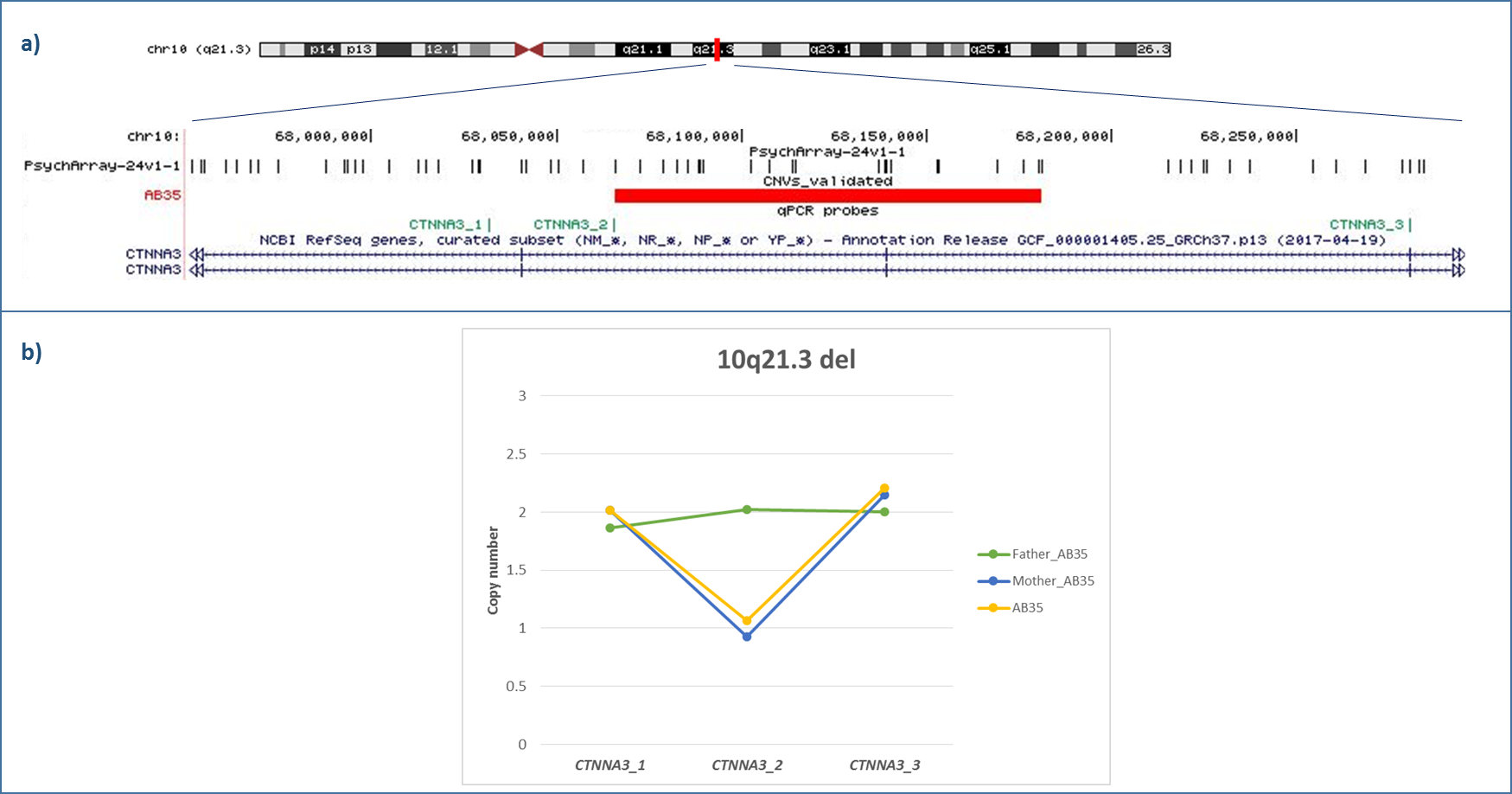


**Supplementary Figure S1_17**


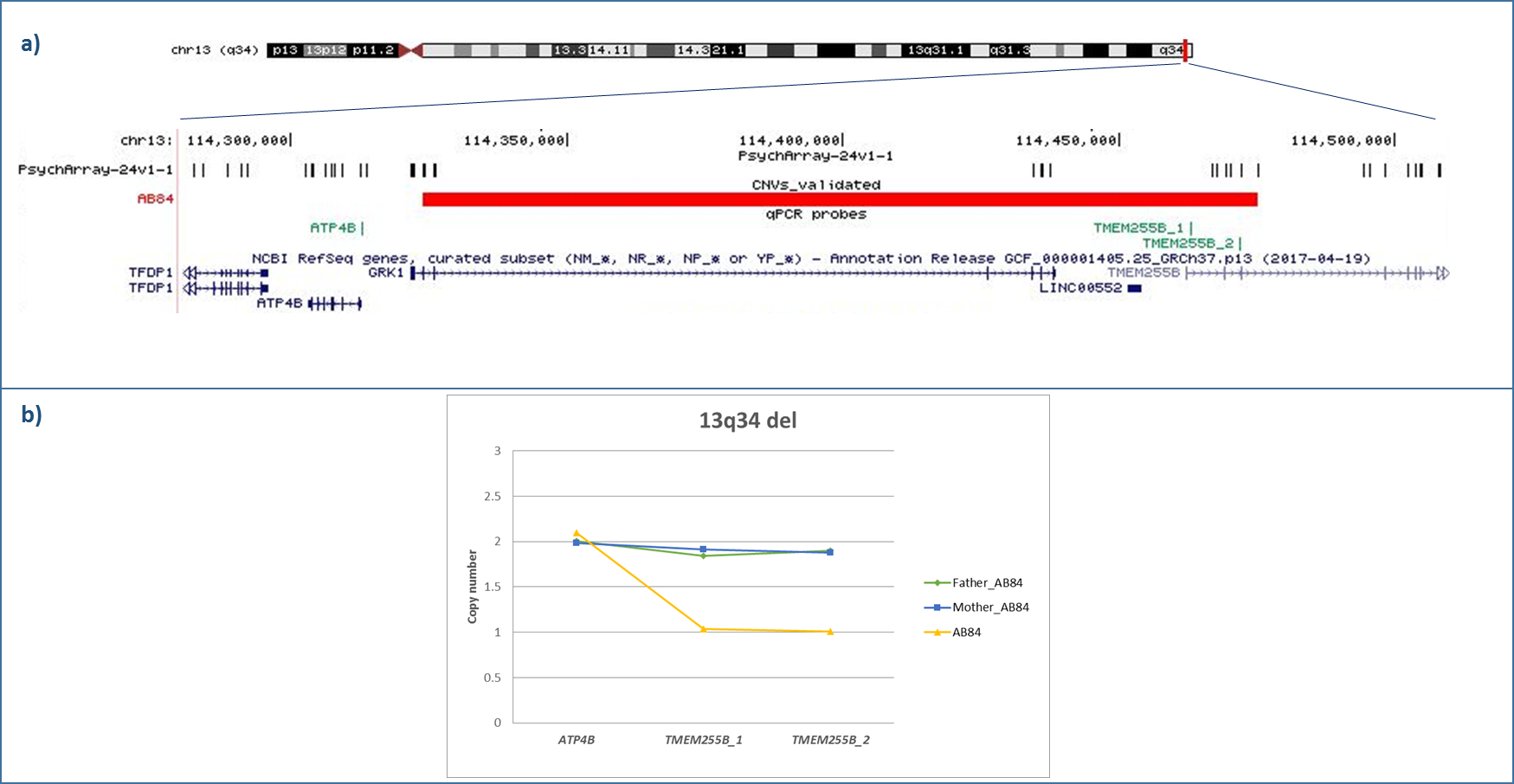


**Supplementary Figure S1_18**


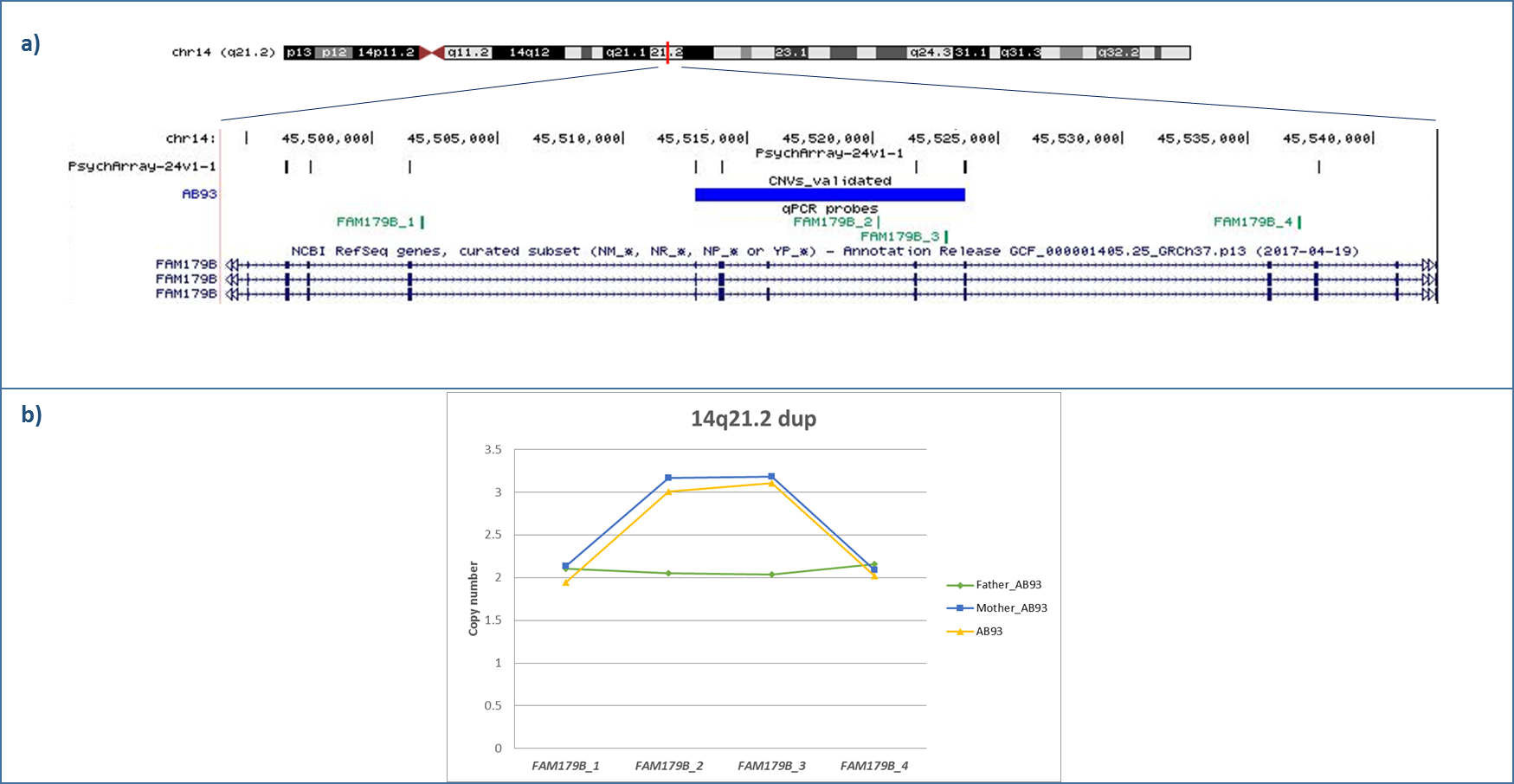


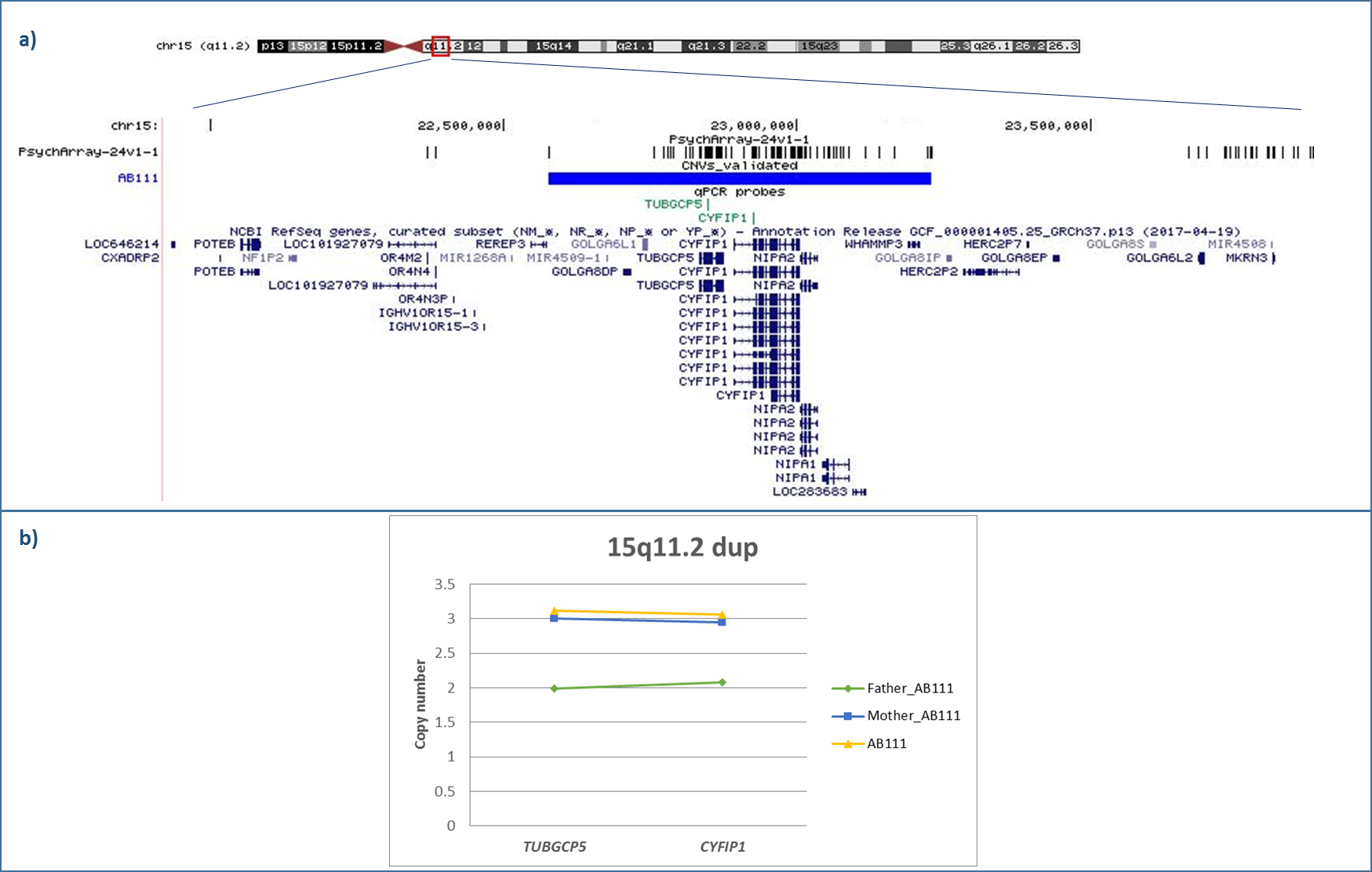
**Supplementary Figure S1_19**


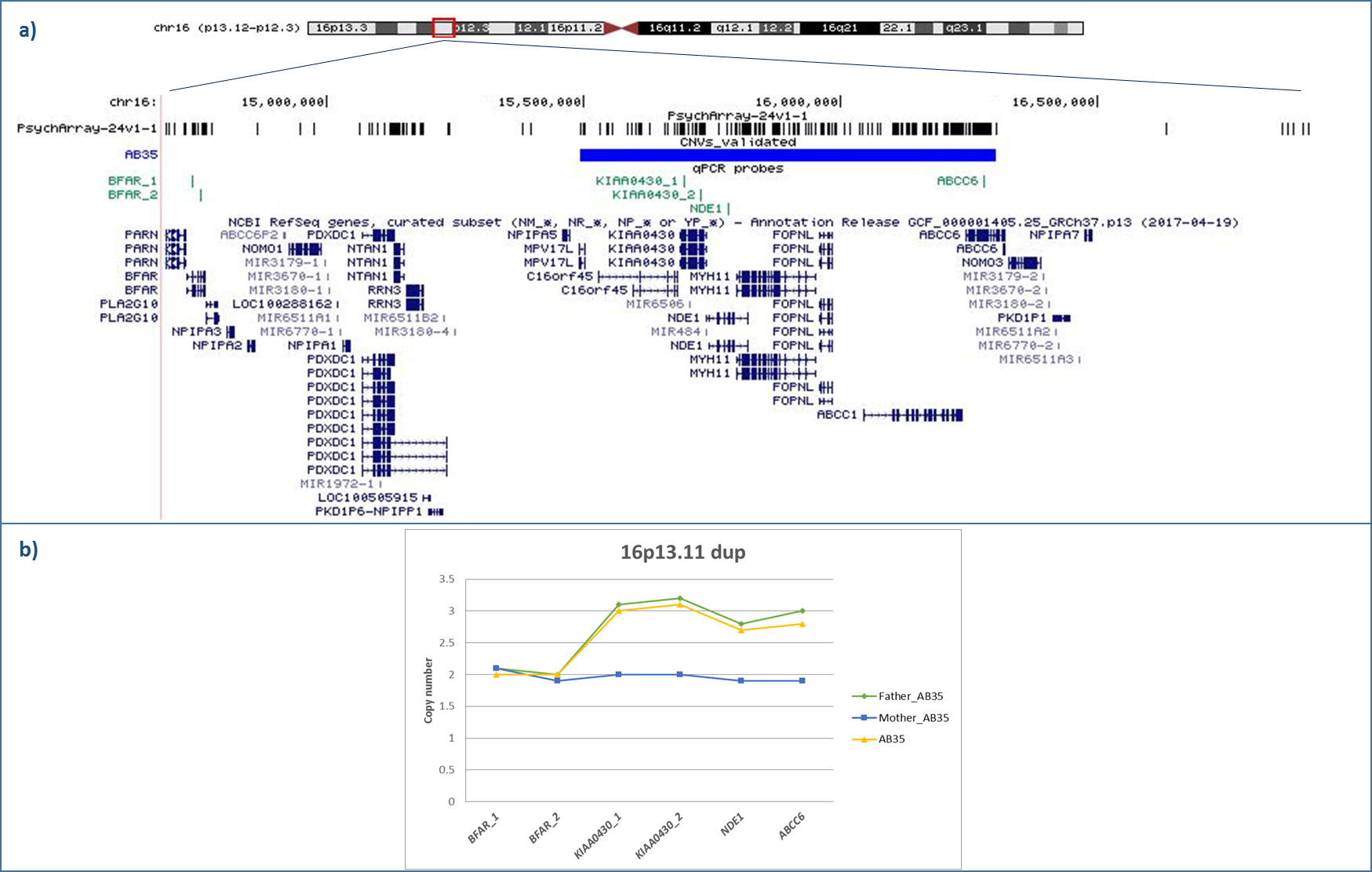
**Supplementary Figure S1_20**

**Supplementary Figure S1_21**


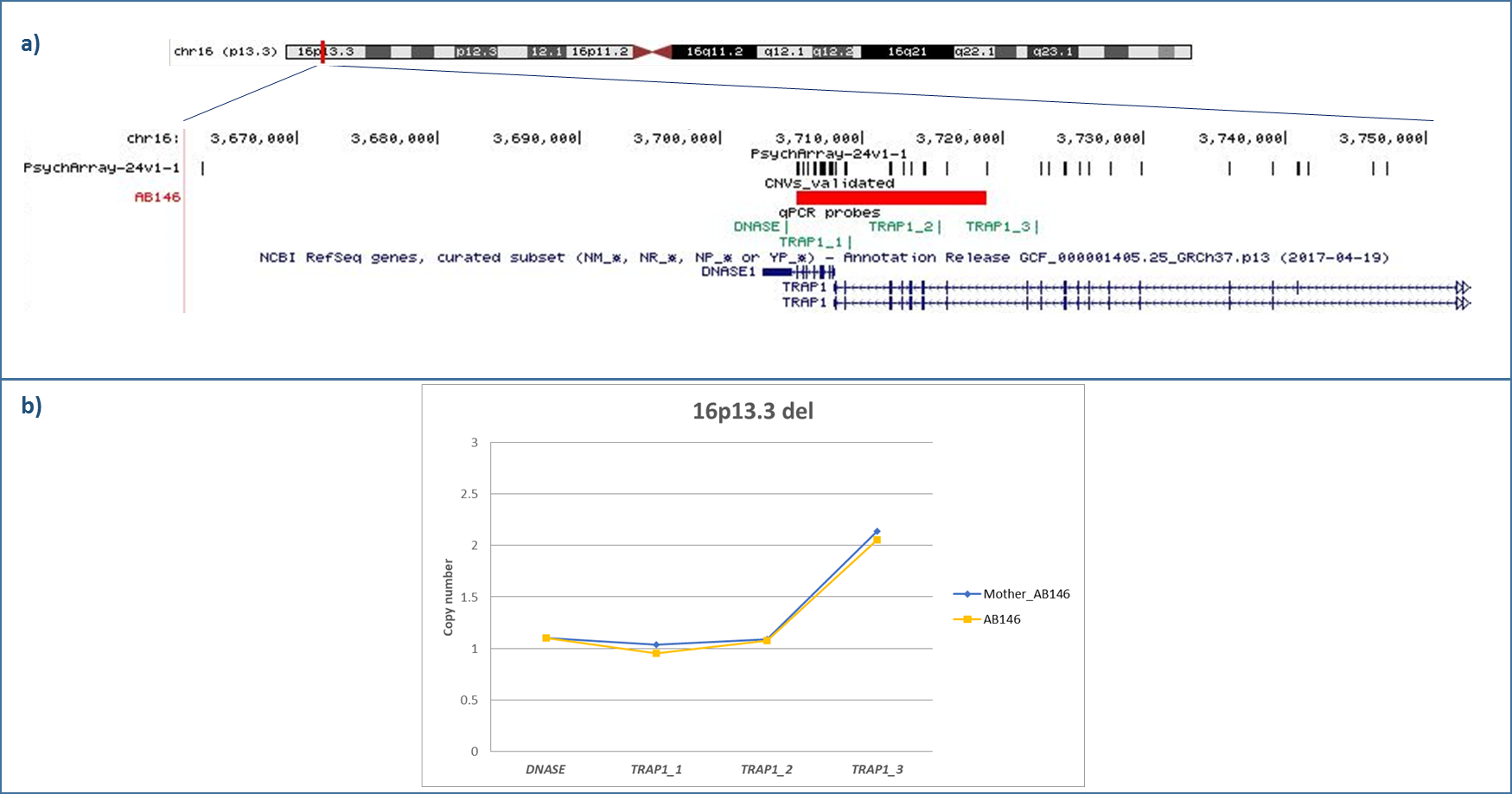


**Supplementary Figure S1_22**


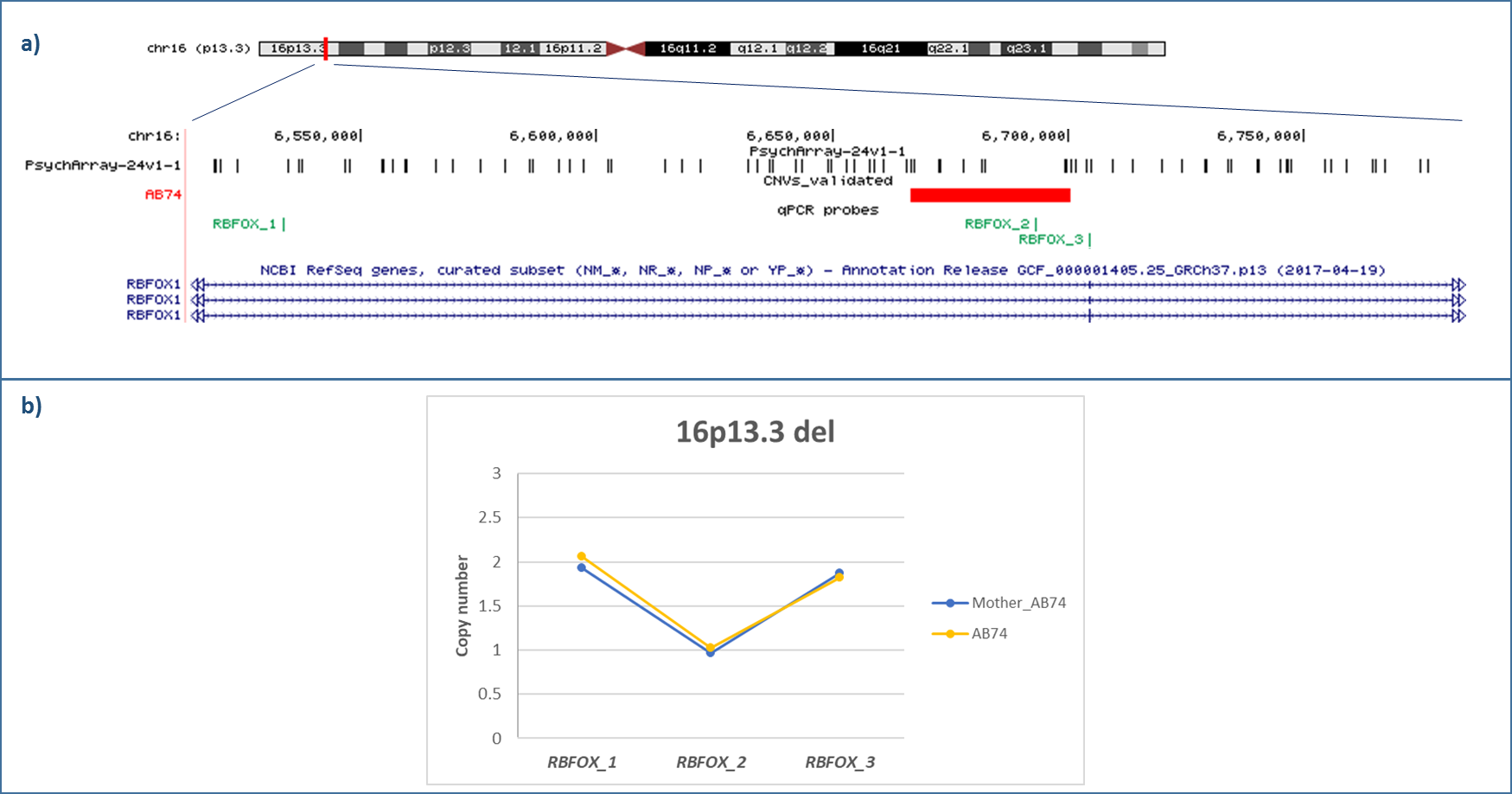


**Supplementary Figure S1_23**
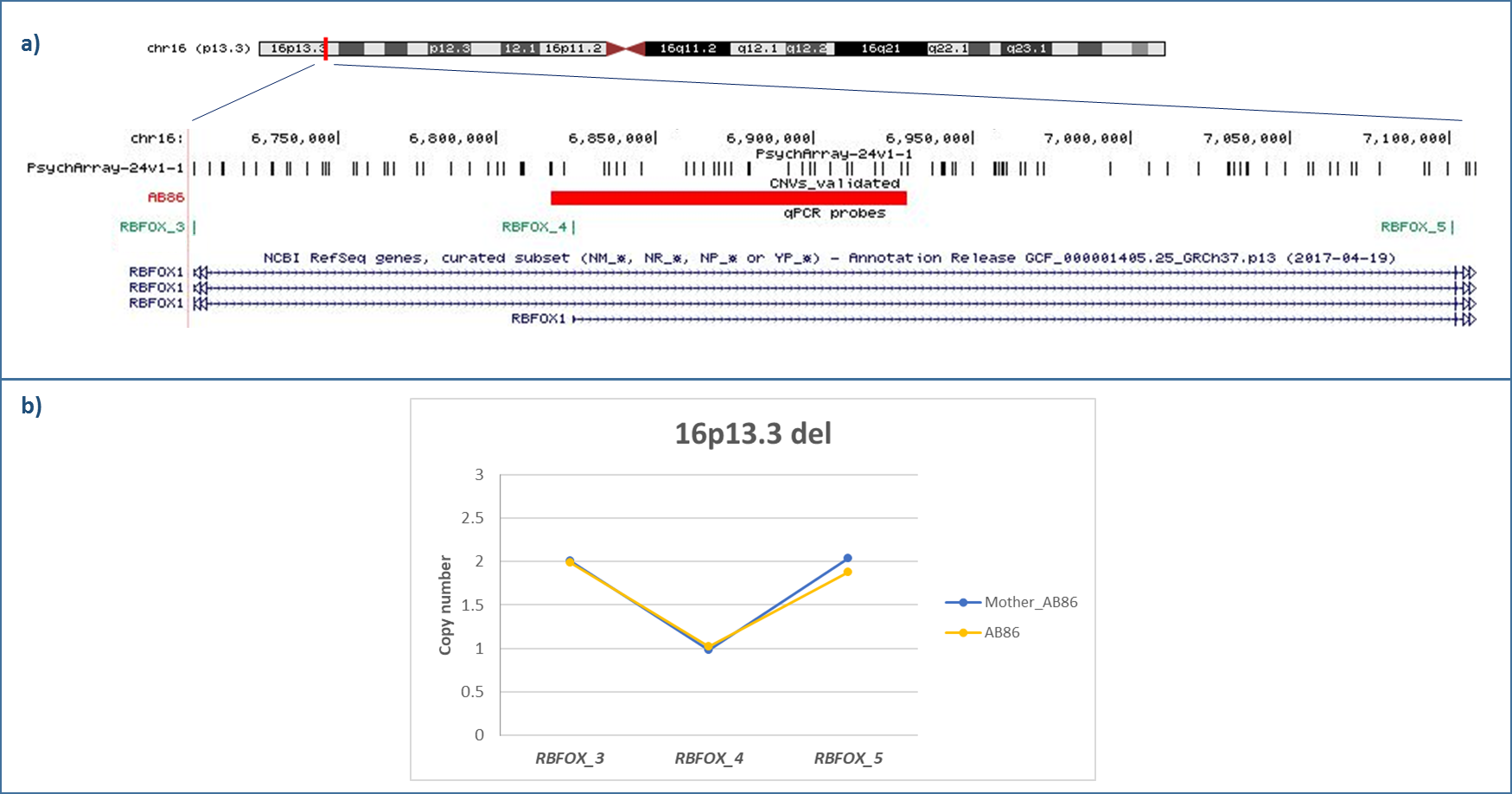


**Supplementary Figure S1_24**


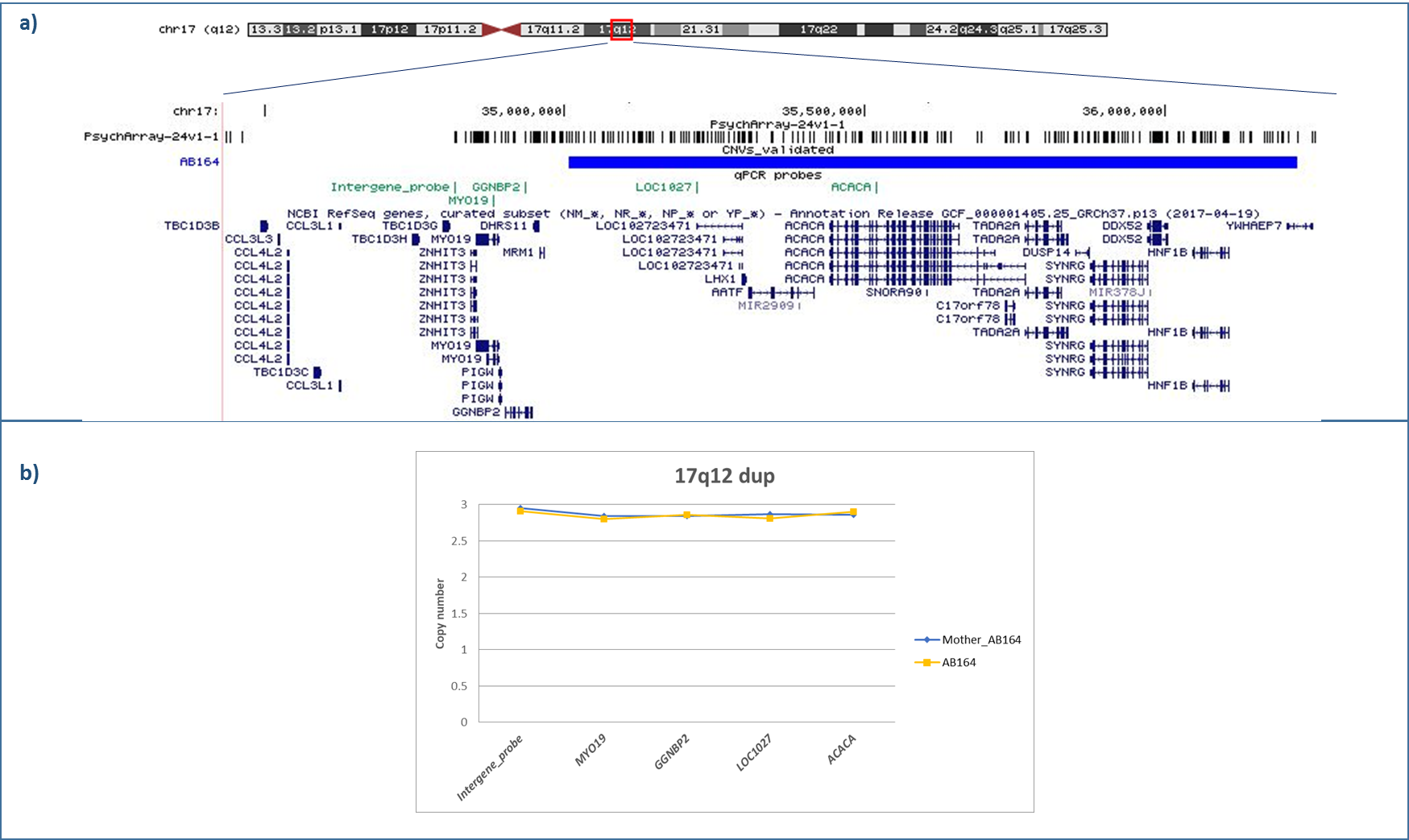


**Supplementary Figure S1_25**


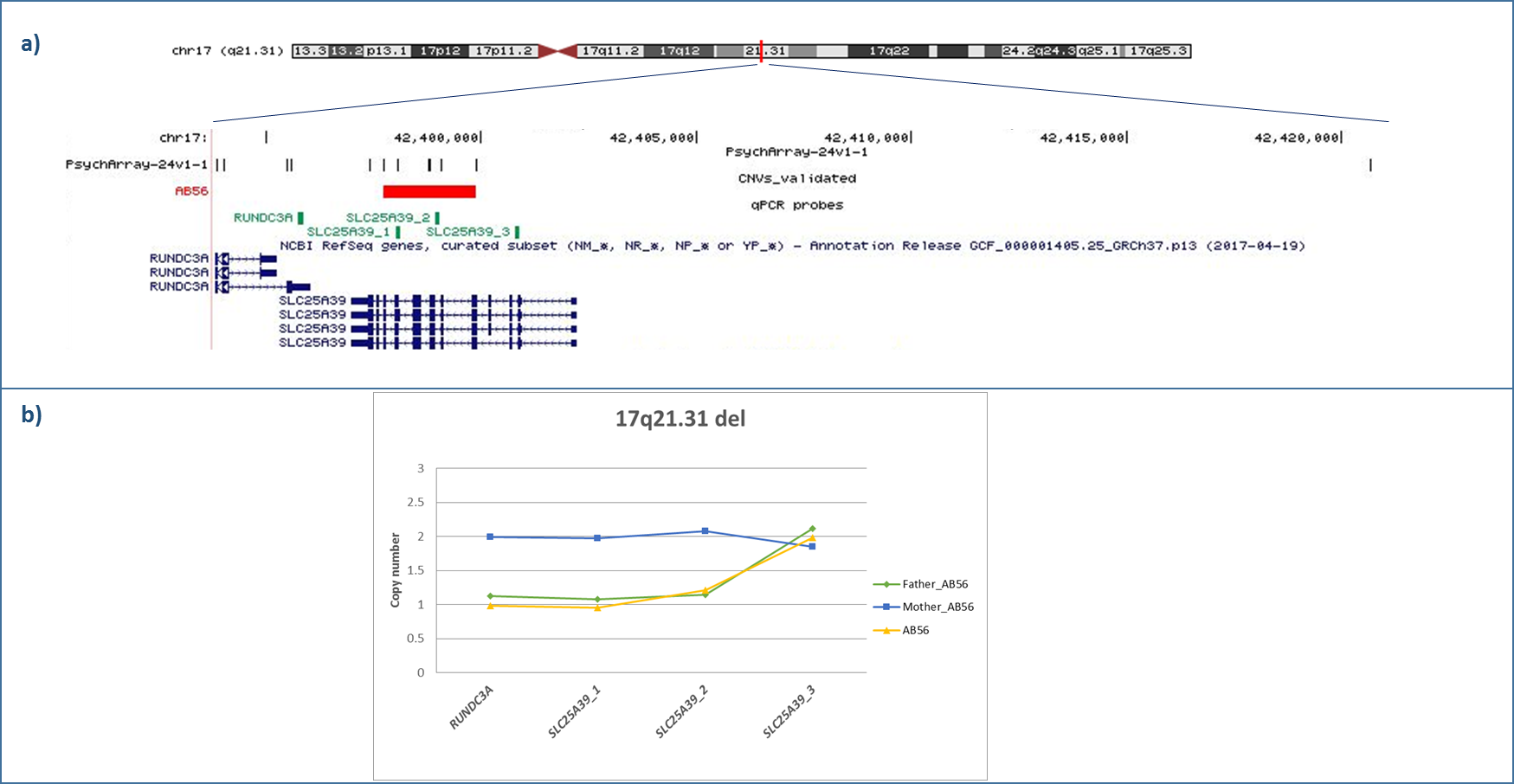


**Supplementary Figure S1_26**


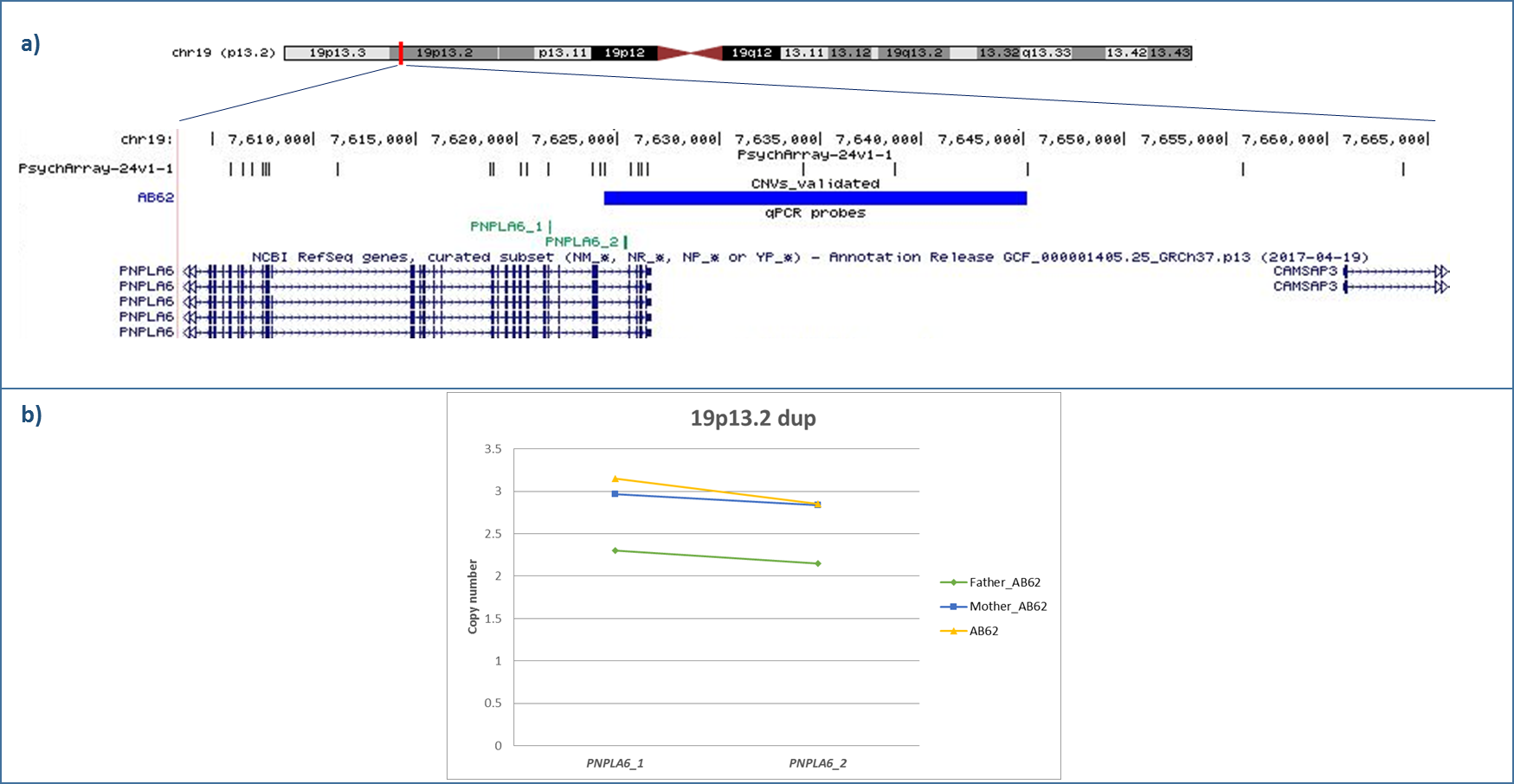


**Supplementary Figure S1_27**


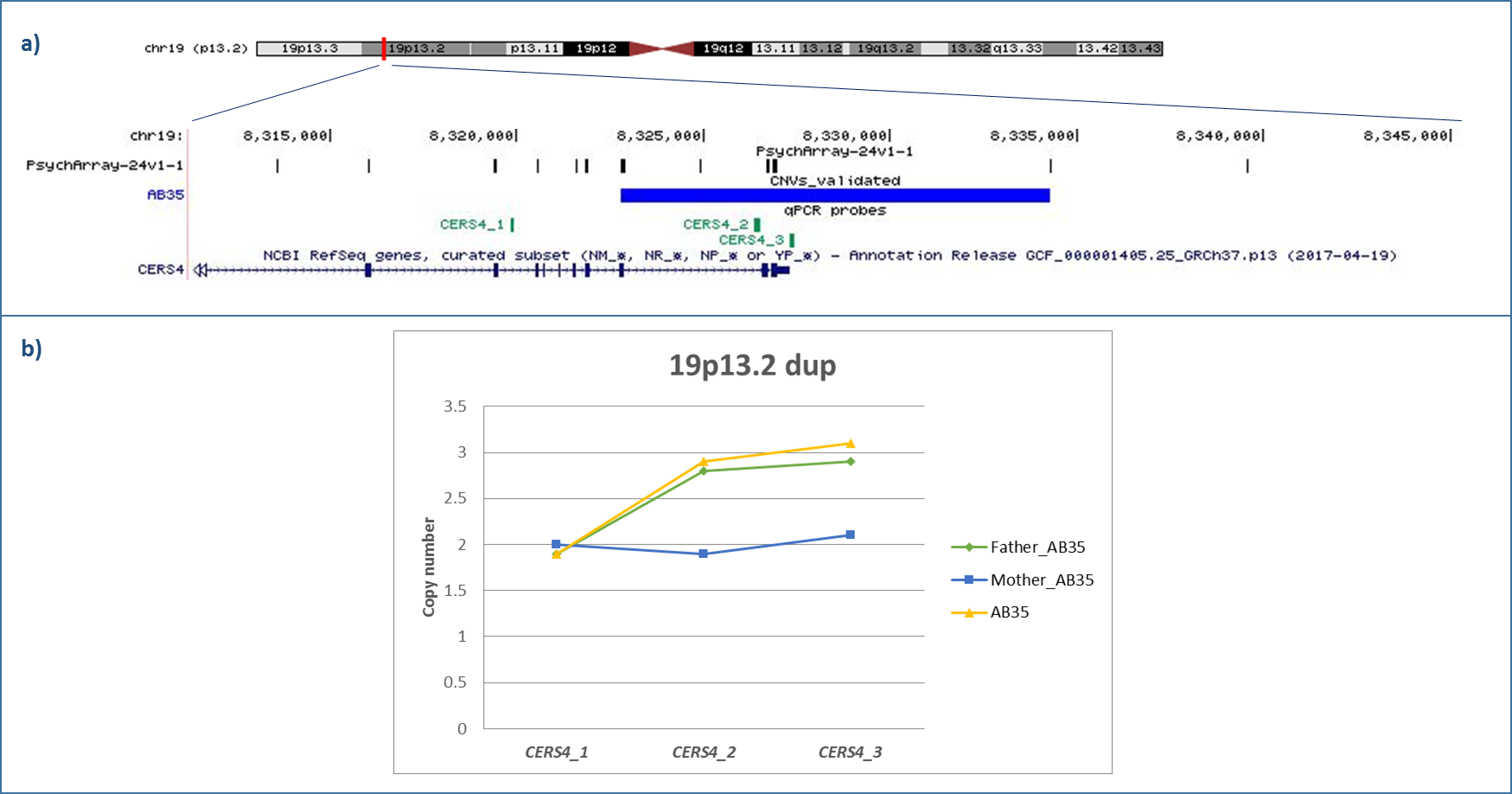


**Supplementary Figure S1_28**


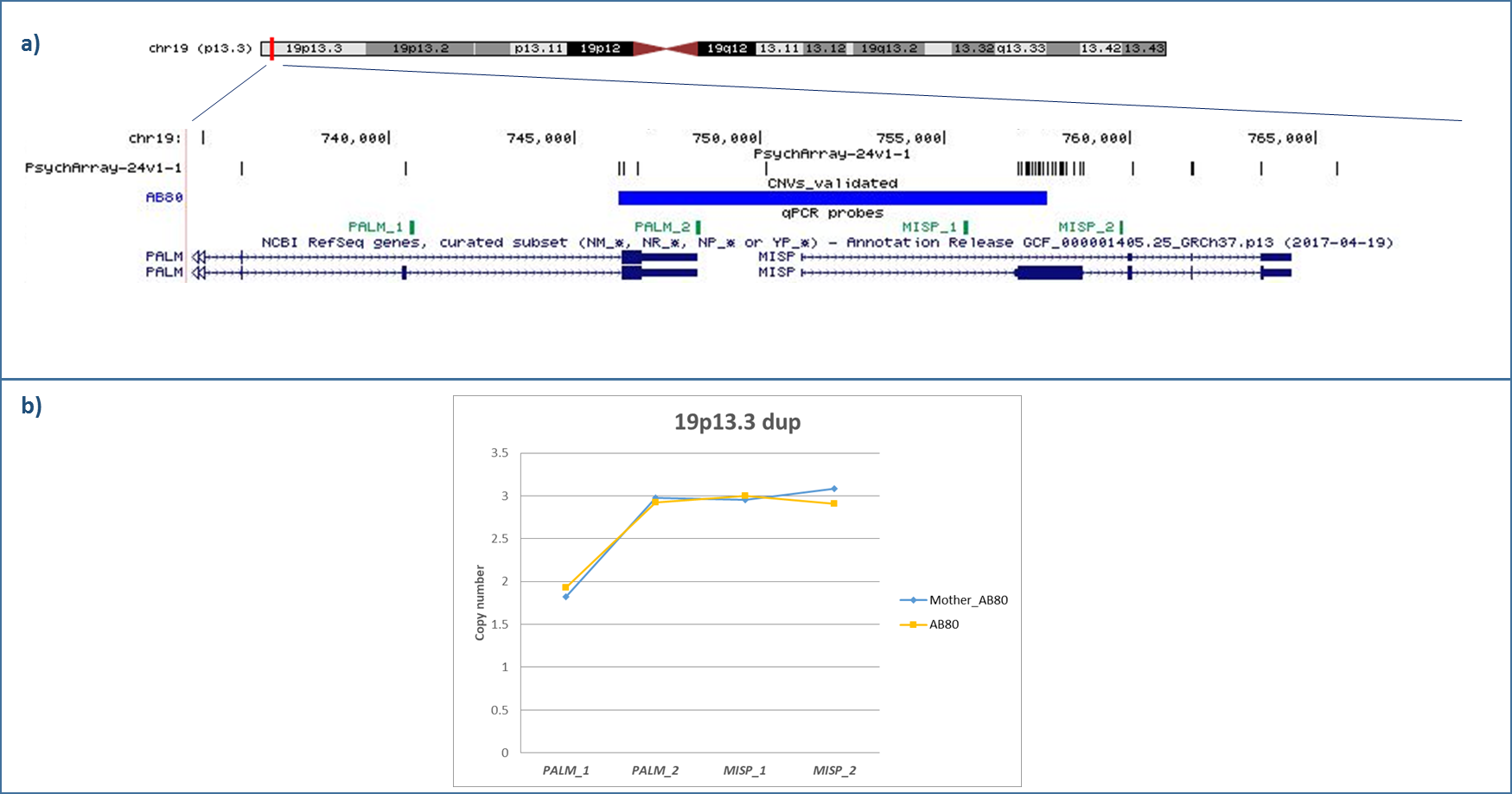


**Supplementary Figure S1_29**


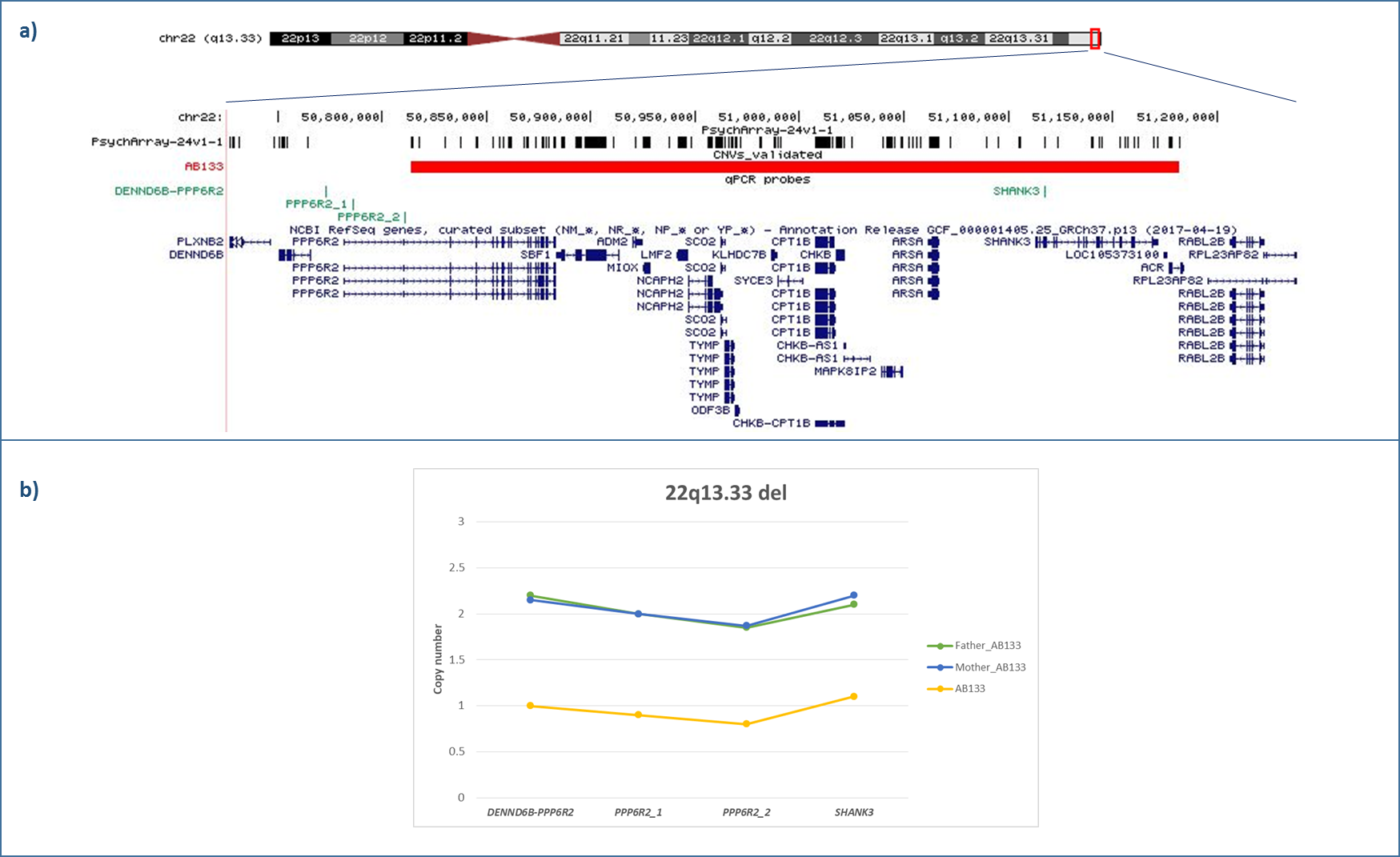


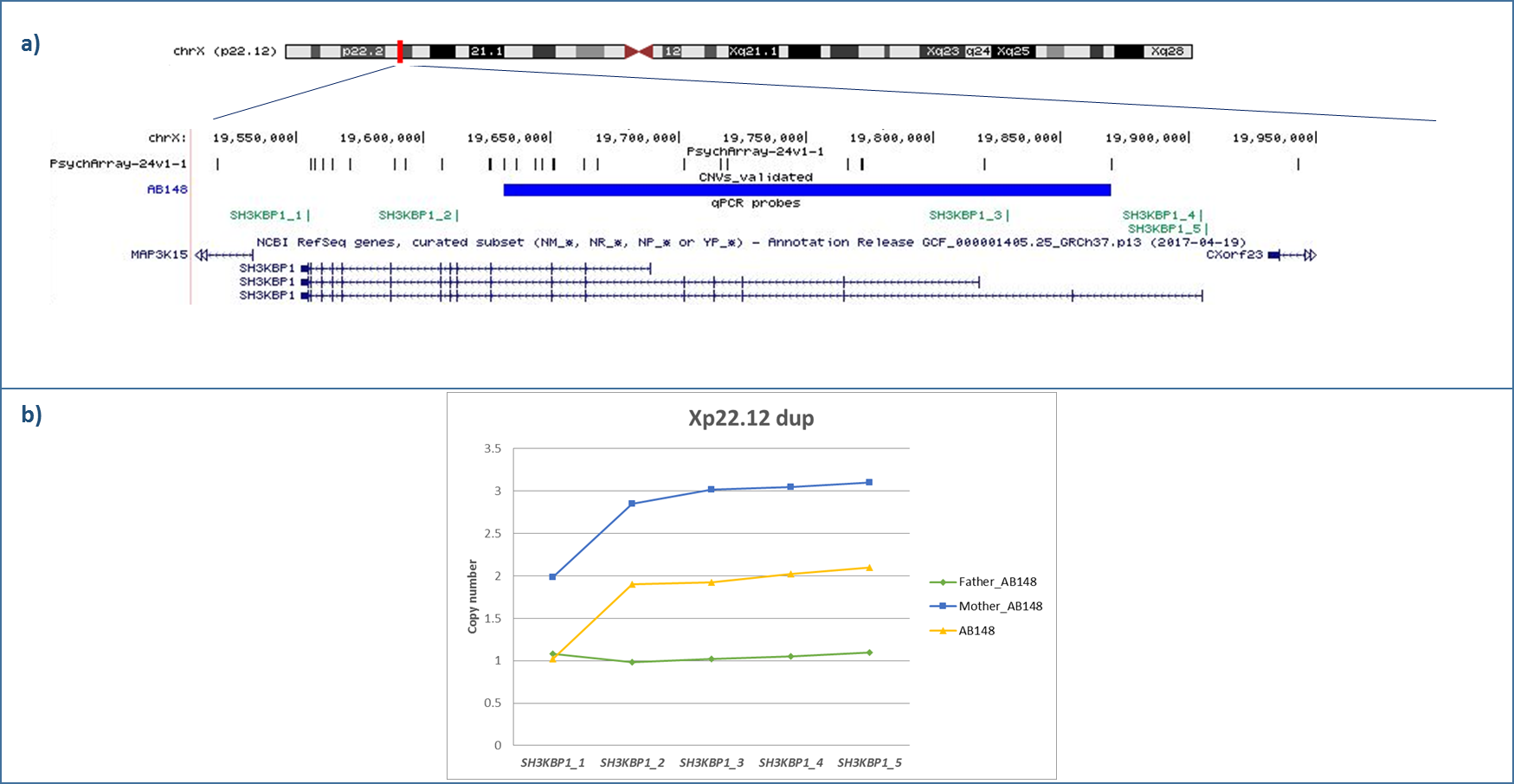
**Supplementary Figure S1_30**
